# Local immune cell contributions to fracture healing in aged individuals – A novel role for interleukin 22

**DOI:** 10.1038/s12276-022-00834-9

**Published:** 2022-08-26

**Authors:** Christian H. Bucher, Julia C. Berkmann, Lisa-Marie Burkhardt, Carolin Paschke, Claudia Schlundt, Annemarie Lang, Angelique Wolter, Alexandra Damerau, Sven Geissler, Hans-Dieter Volk, Georg N. Duda, Katharina Schmidt-Bleek

**Affiliations:** 1 Julius Wolff Institute of Biomechanics and Musculoskeletal Regeneration, Charité - Universitätsmedizin Berlin, corporate member of Freie Universität Berlin and Humboldt-Universität zu Berlin, Berlin, Augustenburger Platz 1, 13353 Germany; 2 BIH Center for Regenerative Therapies, Berlin Institute of Health at Charité - Universitätsmedizin Berlin, Charitéplatz 1, 10117 Berlin, Germany; 3 Berlin-Brandenburg School for Regenerative Therapies, Charité - Universitätsmedizin Berlin, corporate member of Freie Universität Berlin and Humboldt-Universität zu Berlin, Augustenburger Platz 1, 13353 Berlin, Germany; 4 Department of Rheumatology and Clinical Immunology, Charité - Universitätsmedizin Berlin, corporate member of Freie Universität Berlin and Humboldt-Universität zu Berlin, Charitéplatz 1, 10117 Berlin, Germany; 5 German Rheumatism Research Centre (DRFZ) Berlin, a Leibniz Institute, Charitéplatz 1, 10117 Berlin, Germany; 6 Institute for Medical Immunology, Charité - Universitätsmedizin Berlin, corporate member of Freie Universität Berlin and Humboldt-Universität zu Berlin, Augustenburger Platz 1, 13353 Berlin, Germany

**Keywords:** Trauma, Mechanisms of disease, Interleukins, Osteoimmunology

## Abstract

With increasing age, the risk of bone fractures increases while regenerative capacity decreases. This variation in healing potential appears to be linked to adaptive immunity, but the underlying mechanism is still unknown. This study sheds light on immunoaging/inflammaging, which impacts regenerative processes in aging individuals. In an aged preclinical model system, different levels of immunoaging were analyzed to identify key factors that connect immunoaged/inflammaged conditions with bone formation after long bone fracture. Immunological facets, progenitor cells, the microbiome, and confounders were monitored locally at the injury site and systemically in relation to healing outcomes in 12-month-old mice with distinct individual levels of immunoaging. Bone tissue formation during healing was delayed in the immunoaged group and could be associated with significant changes in cytokine levels. A prolonged and amplified pro-inflammatory reaction was caused by upregulated immune cell activation markers, increased chemokine receptor availability and a lack of inhibitory signaling. In immunoaged mice, interleukin-22 was identified as a core cell signaling protein that played a central role in delayed healing. Therapeutic neutralization of IL-22 reversed this specific immunoaging-related disturbed healing. Immunoaging was found to be an influencing factor of decreased regenerative capacity in aged individuals. Furthermore, a novel therapeutic strategy of neutralizing IL-22 may successfully rejuvenate healing in individuals with advanced immune experiences.

## Aging: immune protein’s role in delayed bone fracture healing

Neutralizing a key cytokine, a signaling protein affecting the immune system could rejuvenate the healing process following prolonged inflammatory responses to bone fractures in elderly patients. Healing patterns vary widely in the elderly following injuries such as bone fractures, and scientists now believe that a patient’s individual innate and adaptive immune profile directly affects the healing process. A short-lived pro-inflammatory response is needed to kickstart healthy healing, but a longer-lasting response can be damaging. In experiments on aged mouse models, the team led by Katharina Schmidt-Bleek at the Julius Wolff Institute in Berlin, Germany, demonstrated that high levels of the cytokine interleukin-22 impaired bone regeneration. Elevated interleukin-22 levels resulted from chronically elevated inflammation and inflammaging, prevalent in elderly patients. The team treated the mice to neutralize interleukin-22, which accelerated the healing process.

## Introduction

Fracture healing outcomes in aged individuals are frequently unsatisfactory, resulting in prolonged immobility and a lack of independent living, associated with a significant reduction in the quality of life. Delayed healing in aged individuals presents clinical challenges and represents a substantial financial burden for society^1–4^.

Healing capacity appears to be highly variable. In some elderly patients, healing responses are poor and prolonged rehabilitation is required, while in others, rapid healing success occurs. The survival probability after bone fracture in aged patients depends on sex and the type of fracture: Hip fractures display the lowest survival probability, at only ~20% in men over 80 years and ~40% in women over 80 years^5,6^. While the prevalence of musculoskeletal conditions varies by age and diagnosis, 20–33% of people across the globe suffer from musculoskeletal injuries or degeneration^7,8^. Especially in elderly individuals, the incidence rates of musculoskeletal injuries, specifically fractures, continue to increase due to the rapidly growing percentage of aged people in industrialized countries. In parallel, these older individuals remain very active (newly shaped recreation patterns), which further increases fracture incidence^9,10^. Current fracture treatment strategies generally do not account for patient age and the potentially reduced healing capacity. These treatment strategies classify patients’ therapy by fracture location, geometry, or soft tissue involvement but otherwise result in identical treatments for the same fracture type, whether the patient is young or old. Hence, repair-augmenting strategies specifically designed for elderly patients could become increasingly important.

The onset of bone healing strongly depends on the initial inflammatory response to the fracture^11^. Healing is initiated by a pro-inflammatory response that activates and attracts cells from the surrounding tissue^12,13^. Timely resolution of this pro-inflammatory phase is essential for successful healing, and consequently, a prolonged pro-inflammatory process may significantly delay the healing process^14^. In previous studies, we showed that the individual immune profile, especially the immunoaging of adaptive immunity, significantly impacts fracture healing potential^15,16^. The local immune phenotype and the immune cells attracted to the site of injury alter signaling cascades. Consequently, the duration, intensity, and composition of the initial pro-inflammatory response to fracture and its counteracting anti-inflammatory reaction have a significant impact on the subsequent healing cascade and ultimately cause variations in healing outcomes.

The initial inflammatory period during healing is defined by innate and adaptive immune responses. Both innate and adaptive immune responses undergo age-related alterations due to changes in the numbers and functions of the cells engaged. Cells of the innate immune system predominantly undergo chronological age-dependent changes, resulting in a myeloid shift, as seen in the aged population, with hematopoietic stem cells favoring the myeloid lineage over the lymphoid lineage^17,18^. Furthermore, multiple studies have shown that innate immune cells, such as neutrophils, monocytes, macrophages, dendritic cells, and NK cells, display impaired receptor expression, chemotaxis, phagocytosis, antigen presentation, cytotoxicity, reactive oxygen species (ROS), and cytokine production upon aging^19,20^. Memory formation in adaptive immunity, however, does not necessarily correlate with the chronological age of the patient since the generation of reactive effector and memory cells depends on exogenous antigen exposure throughout life. Thus, the adaptive immune profile reflects the individual immune experience rather than chronological aging. Adaptive immune cells significantly shift in memory/effector subpopulations, resulting in a reduction in the naïve cell pool and the accumulation of late-differentiated effector and memory cells with prolonged or repetitive antigen exposure. In particular, effector T cells can migrate to nonimmune tissues, with a preference for inflamed sites and show a heightened state of basal inflammation and excessive inflammatory responses compared to younger, more naïve immune cells^21^. Thus, increased immune experience creates a slightly pro-inflammatory cytokine and chemokine milieu, which is termed inflammaging. Inflammaging describes a state of sterile, chronic, and systemic low-grade inflammation^22,23^. Inflammaging has only recently been acknowledged as a factor underlying most age-related pathologies. An inflammaged cytokine milieu in homeostasis and an increased pro-inflammatory response upon activation are hallmarks of the age-related decline in regenerative capacity.

Age-associated functional changes in the immune system and inflammaging alter many processes involved in tissue homeostasis, such as metabolism, vascularization, and bone remodeling^24–26^. Increased adaptive immune experience alters osteoblastic, osteoclastic, and progenitor cell behavior in intact bone^25^. However, immunoaging—the integration of age-associated and inflammaging-associated changes—also refers to the individual responses of tissues to injury. Understanding the principles underlying immunoaging during fracture healing would create new opportunities to personalize fracture treatment.

Here, we hypothesize that rejuvenation of the immunoaged response to fracture would accelerate the regeneration process in an aging organism. We dissected the contributions of high- and low-experienced adaptive immune responses in aged mice during long bone fracture healing. Comprehensive characterization of the model was performed using a multidimensional approach, including analyses of the microbiome and differences in immune phenotypes, and correlated with healing outcomes after injury. Based on local immune phenotyping and immune cell activation pattern analyses, expressed cytokines and reactive oxygen species (ROS) were identified and analyzed for their impact on mesenchymal precursor subsets. In doing so, we differentiated two “aging” phenomena and identified an essential signaling pathway that appears to control healing in immunoaged individuals. Finally, we applied a therapeutic approach to rescue the diminished healing capacity in immunoaged individuals in the context of bone regeneration.

## Materials and methods

### Animal housing and surgery

Female C57BL/6NCrl mice were purchased from Charles River, Germany, at the age of 6 weeks. Female mice were used in this study, as female mice have a slower fracture healing process than male mice^27^. An improvement in the healing process can be seen in a more accurately quantifiable and controllable way after 21 days of healing, and female murine bone regeneration may further benefit from treatment strategies^28^. The mice were housed under specific pathogen-free (SPF) conditions or under exposed housing conditions. SPF housing conditions included individually ventilated cages and air filtration, and exposed housing was achieved by reducing the filtration of the air supply. The light/dark cycle was set to a 12 h rhythm, the temperature was set and maintained at 20 °C, and water and food were offered ad libitum. Mice were housed under different housing conditions until they reached the age of 12 months. One group from each condition underwent cervical dislocation under deep anesthesia, and the organs and tissues were removed for further analysis. The osteotomy groups each received an analgesic and antibiotic (buprenorphine and clindamycin, respectively) and were anesthetized continuously with an isoflurane/oxygen mixture. The mice were kept on a heating pad set to 37 °C, eye ointment was applied to reduce eye dehydration, and the surgical area was shaved and aseptically treated. An incision along the axis of the left femur and blunt preparation of the adjacent muscles were performed by sparing the integrity of ligaments and muscles as much as possible. The femur was exposed with forceps by shielding the sciatic nerve from damage. An external rigid fixator (MouseExFix, RISystem, Davos, Switzerland) was mounted after drilling 0.4 mm holes orthogonal to the femur length and fixation with 4 pins. An osteotomy was introduced between the inner pins of the installed fixator with a Gigli wire saw with a diameter of 0.66 mm to introduce a 0.7 mm osteotomy gap. Muscles and ligaments were repositioned appropriately, the skin was closed with a nonresorbable suture, and the wound was further closed with a spray adhesive. Postanesthesia care was performed via subcutaneous injection of supplemental fluids for faster recovery from fluid deprivation during surgery and heat supplied by infrared lamps. Until 3 days post-surgery, further analgesics (tramadol) were administered via the drinking water, and a soaked food pellet was offered on the cage bottom. Three or 21 days post-surgery, the osteotomized mice underwent cervical dislocation under deep anesthesia as described above for the control groups, and the organs and tissues were removed and prepared for further analysis. All surgeries and animal handling were conducted according to the FELASA guidelines and reported following the ARRIVE guidelines. Animal experiments in the current study were compliant with the German Animal Welfare Act, and ethical approval was granted by the local state authorities (Landesamt für Gesundheit und Soziales Berlin).

### Cell phenotype and functionality analysis

The spleen and bone marrow were converted to a single-cell suspension by flushing the bone marrow and mincing the tissue through a 40 µm nylon mesh filter. The hematoma was collected with the adjacent bone marrow around the fixation pins, which also resembled a hematoma due to the drilling of the bone, using fine forceps and minced through a 40 µm nylon mesh filter. The erythrocytes were lysed with RBC lysis buffer (BioLegend, San Diego, USA). Live/dead discrimination was performed with the Fixable Live/Dead Blue staining kit for UV excitation (Invitrogen, Waltham, USA). Staining with antibodies was performed in flow cytometry buffer (phosphate-buffered saline (PBS) including bovine serum albumin (0.5% w/v) and sodium acid (0.1% w/v) to block the internalization of some surface markers), with constant cooling on ice. Nonspecific binding of antibodies to Fc receptors was blocked with FcX blocking solution (BioLegend, San Diego, USA). A list of all antibodies used in this study can be found in the supplementary material (Supplementary Table 1). Intracellular staining for cytoplasmic targets was performed with fixation and permeabilization kit (BioLegend, San Diego, USA), while intranuclear staining was performed with the TrueNuclear Transcription Factor buffer set (BioLegend, San Diego, USA) according to the manufacturer’s protocol. The stained cells were analyzed on an LSR Fortessa SORP system by applying a bead-based compensation matrix. Gating and analysis of the population were performed with FlowJo software (Tree Star, Ashland, OR, USA) using fluorescence minus one (FMO) controls to set the gates. Gating strategies can be found in the supplementary material, Supplementary Figs. 5-8.

### Cytokine secretion

Blood from all mice was collected by intracardial puncture under deep anesthesia and stored in heparin-coated tubes. The plasma was collected after centrifugation and stored at −80 °C until further use. The following multiplexed fluorescence-labeled immunosorbent assay kits were used according to the manufacturer’s instructions with filter plates: LegendPlex Mouse Cytokine Panel 2, LegendPlex Mouse T Helper Cytokine Panel, and LegendPlex Mouse Proinflammatory Chemokine Panel (all BioLegend, San Diego, CA, USA). The multiplexed bead-based immunoassays were measured on a CytoFlex LX system (BeckmanCoulter, Brea, CA, USA) and analyzed with LegendPlex Software (v8.0, BioLegend, San Diego, CA, USA).

### Microbiome analysis

Mice were sacrificed, and the collected ceca were immediately frozen in liquid nitrogen. Samples were stored at −80 °C until they were shipped on dry ice to the Institute for Food & Health (ZIEL) at the Technical University of Munich, Germany. The cecal content was stored at −80 °C until further RNA processing. 16S rRNA gene tag sequencing (MiSeq, Illumina, San Diego, CA, USA) of the V3–V4 regions was performed at the ZIEL Core Facility Microbiome. Raw sequence reads were processed with IMNGS (www.imngs.org) based on the UPARSE approach, a *de novo* operational taxonomic unit (OTU) selection strategy^29^. OTUs clustered at 97% sequence similarity, and only OTUs with a relative abundance of ≥0.5% total reads in at least one sample were further processed. Additional parameters were max. two barcode mismatches tolerated, ten nucleotides each trimmed at the 5′ and 3′ ends, trim quality score of 3 and 3 expected errors, as well as a read length between 300 and 600. The R package Rhea was used for detailed downstream analysis to assess OTU normalization, relative abundances, and alpha diversity within species based on the Shannon effective number of species^30^.

### Reactive oxygen species (ROS) analysis

Isolated bone marrow cells were resuspended at a final concentration of 500,000 cells/ml in RPMI 1640 with stable l-glutamine (Biochrom, Berlin, Germany) supplemented with 10% heat-inactivated FBS (Thermo Fisher, Waltham, MA, USA) and penicillin (100 U/ml) and streptomycin (0.1 mg/ml, Biochrom, Berlin, Germany). The intracellular ROS levels were analyzed using a CellROX Flow Cytometry assay, which was performed at 37 °C using the CellROX Deep Red Flow Cytometry Assay Kit (Thermo Fisher, Waltham, MA, USA) according to the manufacturer’s instructions. Briefly, cells were treated with 0.5 mM *N*-acetylcysteine (NAC, Sigma–Aldrich, St. Louis, USA) for 1 h, followed by ROS induction using 200 μM tertbutyl hydroperoxide (TBHP, 30 min). ROS staining was conducted via the addition of CellROX Deep Red Reagent (750 nM in DMSO, 30 min), and distinguishing living/dead cells was enabled using Sytox Blue Dead Cell stain solution in DMSO (1 µM, 15 min, addition during the last 15 min of CellROX staining). After staining, the cells were centrifuged, resuspended in 200 µl PBS and filtered through a 35 µm cell strainer (Falcon, Corning, Corning, USA). Flow cytometric measurement was performed at 405 (Sytox) and 635 nm (CellROX Deep Red) excitation wavelengths, and fluorescence emission was collected in the V450-A (Sytox) and R 670-A (CellROX) channels using a BD LSR Fortessa SORP (BD Biosciences, Franklin Lakes, USA).

### Microcomputed X-ray tomography

The osteotomized bones were harvested after cervical dislocation and directly fixed in ice-cold 4% paraformaldehyde/PBS (Electron Microscopy Sciences, Hatfield, PA, USA) for 6 h at 4 °C with continuous shaking. The fixated bones were stabilized within a serological pipette, and the external fixator was removed. The healing outcome was monitored via microcomputed tomography (µCT) with a SkyScan 1172 (Bruker, Kontich, Belgium). The voxel size was set at 8 µm, and source energy of 70 kV and 142 µA was applied to scan the bones with beam filtering through a 0.5 mm aluminum filter. The shadow images were reconstructed using a modified Feldkamp algorithm implemented in the software suite NRecon (Bruker, Kontich, Belgium) by applying ring artifact reduction and beam hardening corrections as specified by control scans. The reconstructed images underwent 2D and 3D analysis using CTan software, and 3D visualization was achieved with CTvox software (both Bruker, Kontich, Belgium). The analysis of the fracture gap included a step for binarization, where a global threshold of 580 mg HA/cm³ was set (standardized by measuring hydroxyapatite (HA) phantoms with known concentrations) and the trabecular structures of the fracture callus were binarized using an adaptive threshold method. The newly formed bone was quantified by excluding the old cortical bone within the callus. This was achieved by using a morphological escalator, a new technique for automatic trabecular-cortical separation. Bone parameters are reported according to the ASMBR guidelines for the assessment of bone microstructure in rodents^31^.

### Progenitor cell differentiation assay

Human bone marrow stromal/stem cells (MSCs) were kindly provided by the Cell and Tissue Harvesting core unit of the BIH Center for Regenerative Therapies (BCRT) and frozen at passage 2. All MSCs were harvested from bone marrow aspirations from patients undergoing hip replacement at Charité - Universitätsmedizin Berlin. Written informed consent was given, and ethics approval was obtained from the local ethics committee/institutional review board (IRB) of the Charité - Universitätsmedizin Berlin. The donor age ranged from 48 to 75 years, with two donors being male and three donors being female. MSCs were thawed and expanded in Dulbecco’s modified Eagle’s medium (DMEM, Thermo Fisher, Waltham, MA, USA) containing 1 g/l glucose and 110 mg/l sodium pyruvate supplemented with 10% fetal bovine serum (FBS Superior, Biochrom, Berlin, Germany) and 1% penicillin–streptomycin (100 units/mL penicillin and 100 µg/mL streptomycin, Thermo Fisher, Waltham, MA, USA). Cells at passage 2+2 were used throughout the study and seeded in tissue culture-treated multiwell plates (Corning, Corning, NY, USA). Osteogenic differentiation was achieved by culturing the MSCs in StemXVivo Osteogenic/Adipogenic Base Medium supplemented with StemXVivo Human Osteogenic Supplement (both R&D Systems, Minneapolis, MN, USA) according to the manufacturer’s instructions for 14 days, with medium exchange twice per week. After culture for 14 days, the cells were fixed with 4% paraformaldehyde/PBS (Electron Microscopy Sciences, Hatfield, PA, USA), stained with 0.5% w/v Alizarin Red S (Sigma-Aldrich, St. Louis, MO, USA), dissolved in dH_2_O and counterstained with 4′,6-diamidin-2-phenylindol (DAPI, Sigma-Aldrich, St. Louis, MO, USA). DAPI fluorescence was measured with an Infinite 200 PRO plate reader (Tecan, Männedorf, Switzerland) using multiple reads per well (MRW) to ensure unbiased signal measurement throughout the well area. A standard curve for interpreting cell numbers was generated by seeding defined cell numbers and directly staining with DAPI after cell attachment to enable normalization of fluorescence and absorbance signals to the cell number. Alizarin Red S absorbance was measured with an Infinite 200 PRO plate reader (Tecan, Männedorf, Switzerland) after dissolving the bound dye in the culture plate with 10% wt/vol cetylpyridinium chloride (Sigma-Aldrich, St. Louis, MO, USA). Adipogenic differentiation was achieved by culturing the MSCs in StemXVivo Osteogenic/Adipogenic Base Media supplemented with StemXVivo Human Adipogenic Supplement (both R&D Systems, Minneapolis, MN, USA) according to the manufacturer’s instructions for 14 days, with medium exchange twice per week. After culture for 14 days, the cells were fixed with 4% paraformaldehyde/PBS (Electron Microscopy Sciences, Hatfield, PA, USA), stained with 2 µg/ml Nile Red (Sigma-Aldrich, St. Louis, MO, USA) predissolved in acetone, diluted in PBS and counterstained with DAPI. Both fluorescence signals were measured, and the DAPI signal was interpreted similarly to the osteogenic differentiation assay for the normalization of cell numbers. Chondrogenic differentiation was achieved by culturing the MSCs as a cell pellet in StemXVivo Chondrogenic Base Medium supplemented with StemXVivo Human Chondrogenic Supplement (both R&D Systems, Minneapolis, MN, USA) according to the manufacturer’s instructions for 21 days, with medium exchange twice per week. After culture for 21 days, the cell pellet was directly dissolved with 125 µg/ml papain from papaya latex (Sigma-Aldrich, St. Louis, MO, USA) in a buffered solution containing 5 mM l-cysteine (Sigma-Aldrich, St. Louis, MO, USA) overnight at 60 °C on a Thermomixer (VWR International, Radnor, PA, USA). The dissolved cell pellets were stored at −20 °C until further use. The glycosaminoglycan (GAG) content was measured with 16 mg/l 1,9-dimethyl-methylene blue (DMMB, Sigma-Aldrich, St. Louis, MO, USA) in a buffered solution and interpreted by dissolving known concentrations of chondroitin sulfate A from bovine trachea (Sigma-Aldrich, St. Louis, MO, USA). To normalize the GAG content to the cell number, a DNA quantification assay was performed to achieve a ratio of GAG/DNA. DNA was quantified using the DNA Quantitation Kit, Fluorescence Assay (Sigma-Aldrich, St. Louis, MO, USA), according to the manufacturer’s instructions. For all assays, representative images were captured with a BZ-X810 microscope (Keyence, Osaka, Japan).

### Scratch assay

MSCs were prepared as described above for the progenitor cell differentiation assay and used at passages 2+2. MSCs were cultured in multiwell plates until they reached confluency in an expansion medium consisting of DMEM (Thermo Fisher, Waltham, MA, USA) containing 1 g/l glucose and 110 mg/l sodium pyruvate supplemented with 10% fetal bovine serum (FBS Superior, Biochrom, Berlin, Germany), 1% GlutaMAX (Thermo Fisher, Waltham, MA, USA) and 1% penicillin–streptomycin. After reaching confluency, the cells were checked for proliferation, and a scratch was introduced with a 1 ml pipette tip. The wells were rinsed and refilled with fresh expansion medium. Wound closure was monitored 6, 18, 24, and 48 h post-injury by capturing phase contrast pictures (BZ-X810, Keyence, Osaka, Japan). Image analysis to assess wound closure was performed using an ImageJ plugin, the wound healing size tool (WHST), kindly provided by ref. ^32^. using Fiji (an open source image processing package based on ImageJ2)^33^.

### Tube formation assay

To assess the ability of cytokines to interfere with the angiogenic potential of endothelial cells, a tube formation assay was performed using human umbilical vein endothelial cells (HUVECs, pooled donors, Lonza, Basel, Switzerland). HUVECs were grown and expanded in an endothelial cell growth medium (EGM-2, Lonza, Basel, Switzerland). Wells were pretreated with a layer of extracellular matrix (Matrigel growth factor reduced, Corning, Corning, NY, USA) for 1 h at 37 °C prior to seeding 240,000 cells/cm^2^ in a 24-well plate. The cells were imaged after 16 h with a BZ-X810 microscope (Keyence, Osaka, Japan), and tube formation was assessed via image-based analysis using Fiji software (an open source image processing package based on ImageJ).

### IL-22 treatment

Mice were osteotomized as described above, and treatment was started 24 h post-surgery to mimic the feasibility of this treatment approach under clinical conditions. The supplementation and neutralization of IL-22 were performed via intraperitoneal injection of recombinant mouse IL-22 (PeproTech, Rocky Hill, NJ, USA) or rat anti-mouse functional grade IL-22 monoclonal antibody (clone IL22JOP, Thermo Fisher, Waltham, MA, USA). IL-22 supplementation was performed with 5 µg protein in 100 µl InVivoPure dilution buffer (BioXCell, Lebanon, NH, USA) every second day, with the last injection timepoint 1 week post-surgery. The neutralizing IL-22 antibody was diluted to 100 µg in 100 µl InVivoPure dilution buffer, with a treatment schedule similar to that described for IL-22 supplementation.

### Visualization and statistics

Statistical analysis and graphical visualization of the data were carried out with GraphPad Prism software (San Diego, CA, USA). All values from in vitro assays are expressed as the mean ± SD, and all values from animal experiments are depicted as the median ± ranges (box-and-whisker plot). An unpaired *t*-test reporting the corresponding two-tailed *p* value was performed where appropriate, including post hoc tests. An unequal variance *t*-test, the Welch *t*-test, was performed for in vivo data, as it can be assumed that the two groups are sampled from Gaussian populations but do not have the same standard deviation. Tukey’s post hoc test was used to exclude outliers where necessary. *P* < 0.05 was considered statistically significant.

## Results

### Advanced preclinical murine model depicting different levels of immunological experience

To distinguish between aging and immunoaging, we analyzed aged mice (12 months old) with two distinct immunological characteristics: mice were housed until the age of 12 months under different housing conditions to achieve distinct immune experiences that were characterized with respect to their microbiota, bone characteristics and immune characteristics. Housing mice with exposure to environmental microorganisms by reducing the filtration of the air supply allowed the immune system to become experienced. Individually ventilated cages (specific pathogen-free housing), however, prevented immune experience, and the immune system remained more naïve.

In detail, exposed housing conditions significantly shifted the subpopulations of lymphocytes, such as depletion of naïve T-cell pools and subsequent accumulation of effector and memory T cells (Table 1). As the two distinct groups of the same age (12 months) differed only in terms of their immunological experience, the groups were termed aged (less-experienced immune system) and immunoaged (experienced immune system). Systemic analysis of the immune cell compartment in the spleen and the bone marrow revealed no significant difference in the proportions of the broadest leukocyte classifications between the two groups (granulocytes, monocytes, NK cells, B and T cells), except for dendritic cells, which were found to be rarer in the bone marrow of the immunoaged group (Table 1). Significant differences were found in the subclassification of lymphocytes in the adaptive immune compartment in the spleen and the bone marrow. Naïve immune cell pools of both the CD4+ and CD8+ T-cell compartments were significantly diminished in the immunoaged group. In parallel, a significantly increased proportion of memory and effector T cells was found in the immunoaged group. The CD4+ T-cell compartment of the immunoaged group showed increased quantities of effector memory (EM, CD62L-CD44+ CD127-KLRG1−) T cells and memory precursor effector (MPEC, CD62L-CD44+ CD127+KLRG1-) T cells: the proportion of CD4+ T_EM_ cells was significantly higher in the immunoaged group than in the aged group (54.3% (±4.6) and 37.3% (±1.2), respectively). The central memory (CM, CD62L+ CD44 + ) population of the CD4+ T-cell pool and short-lived effector cells (SLEC, CD62L-CD44+ CD127-KLRG1+) did not significantly differ between the two groups. The CD8+ T-cell pool showed similar trends, with increased memory formation and diminished naïve (CD62L+ CD44-) T cells. The T_CM_ pool was significantly increased (61.1% (±1.3) of all CD8+ T cells) in the spleen of the immunoaged group compared to the aged group, with only 36.6% (±2.4) CD8+ T_CM_ (Table 1). In the bone marrow, some subpopulations of T cells were more strongly represented, as CD4+ SLECs and CD8+ MPECs were found in larger proportions in the bone marrow of the immunoaged group (Table 1).

**Table 1:** Adaptive immune experience, especially T-cell experience, is significantly increased under exposed housing conditions.

Spleen				Bone marrow			
phenotypic distribution				phenotypic distribution			
**subpopulation**	**aged**	**immunoaged**	***p value***	**subpopulation**	**aged**	**immunoaged**	***p value***
Granulocytes	13.0% (0.9)	14.6% (7.0)	0.71	Granulocytes	68.2% (2.9)	72.8% (1.5)	0.11
Monocytes/Macrophages	12.5% (1.0)	16.2% (2.4)	0.06	Monocytes/Macrophages	13.5% (0.7)	12.5% (0.7)	0.22
B cells	36.4% (3.5)	36.4% (9.6)	1.00	B cells	7.7% (0.6)	7.3% (1.0)	0.67
T cells	31.2% (3.9)	27.1% (0.3)	0.12	T cells	6.7% (1.2)	4.9% (0.2)	0.11
NK cells	2.7% (0.6)	2.2% (0.2)	0.12	NK cells	1.5% (0.3)	1.5% (0.1)	0.93
Dendritic cells	4.1% (0.4)	3.6% (0.3)	0.10	Dendritic cells	2.4% (0.5)	1.0% (0.2)	0.02
**CD4**+ **T cells**				**CD4**+ **T cells**			
**subpopulation**	**aged**	**immunoaged**	***p value***	**subpopulation**	**aged**	**experienced**	***p value***
Naïve	29.6% (6.0)	17.8% (4.3)	0.03	Naïve	10.0% (3.6)	3.0% (0.8)	0.02
CM	6.1% (1.2)	6.3% (0.9)	0.89	CM	7.1% (1.6)	5.3% (1.4)	0.20
EM	37.3% (5.6)	54.3% (4.6)	<0.01	EM	22.6% (3.1)	40.5% (5.8)	<0.01
MPEC	23.8% (1.3)	17.5% (0.5)	<0.01	MPEC	47.8% (1.0)	36.0% (3.9)	<0.01
SLEC	3.8% (1.0)	3.4% (0.2)	0.58	SLEC	9.5% (1.3)	13.4% (1.8)	0.02
**CD8**+ **T cells**				**CD8**+ **T cells**			
**subpopulation**	**aged**	**immunoaged**	***p value***	**subpopulation**	**aged**	**immunoaged**	***p value***
Naïve	50.2% (3.5)	22.3% (2.2)	<0.01	Naïve	24.1% (2.4)	5.8% (1.9)	<0.01
CM	36.6% (2.4)	61.1% (1.3)	<0.01	CM	52.2% (8.0)	70.9% (2.0)	0.01
EM	3.9% (1.4)	6.9% (1.9)	0.07	EM	4.7% (2.3)	7.5% (1.7)	0.14
MPEC	3.5% (0.1)	3.4% (0.9)	0.98	MPEC	6.6% (1.7)	3.5% (0.6)	0.03
SLEC	6.0% (2.2)	5.9% (0.4)	0.92	SLEC	10.8% (4.4)	11.6% (2.4)	0.79

Flow cytometry analysis of the systemic immune phenotype in the spleen and in the bone marrow. There were significant differences in immune cell composition between the spleen and bone marrow. While the dominant populations in the bone marrow were components of the innate immune system, the dominant populations in the spleen were components of the adaptive immune system. No significant differences in the phenotypic distribution were found between the immunoaged and aged groups, except for dendritic cells, which were diminished in the bone marrow of the immunoaged group. However, the proportion of naïve T cells was significantly decreased in the immunoaged group in both compartments. The dominant memory populations among the CD4+ T cells in the spleen were effector memory (EM) T cells, whereas, among the CD8+ T cells, the dominant memory populations were central memory (CM) T cells. Additionally, effector cells in the spleen and the bone marrow, namely, memory precursor effector cells (MPEC) and short-lived effector cells (SLECs), in both the CD4+ and CD8+ T-cell compartments differed according to the experience level. The proportional contributions of immune cell compartments are presented in the table, and *p* values are provided for the comparison between the immunoaged and aged groups. *N* = 4 per group; mean (SD); Welch’s *t*-test.

Overall, we established conditions to generate immunologically experienced (immunoaged) and less-experienced (aged) individuals as a preclinical model in aged mice. The data and characterization illustrate that this advanced model allows us to study immunological diversity and appears to mimic the heterogeneity of immunological experience observed with increased age^34^.

### Fracture healing is disturbed in the immunoaged group

**Fig. 1:**
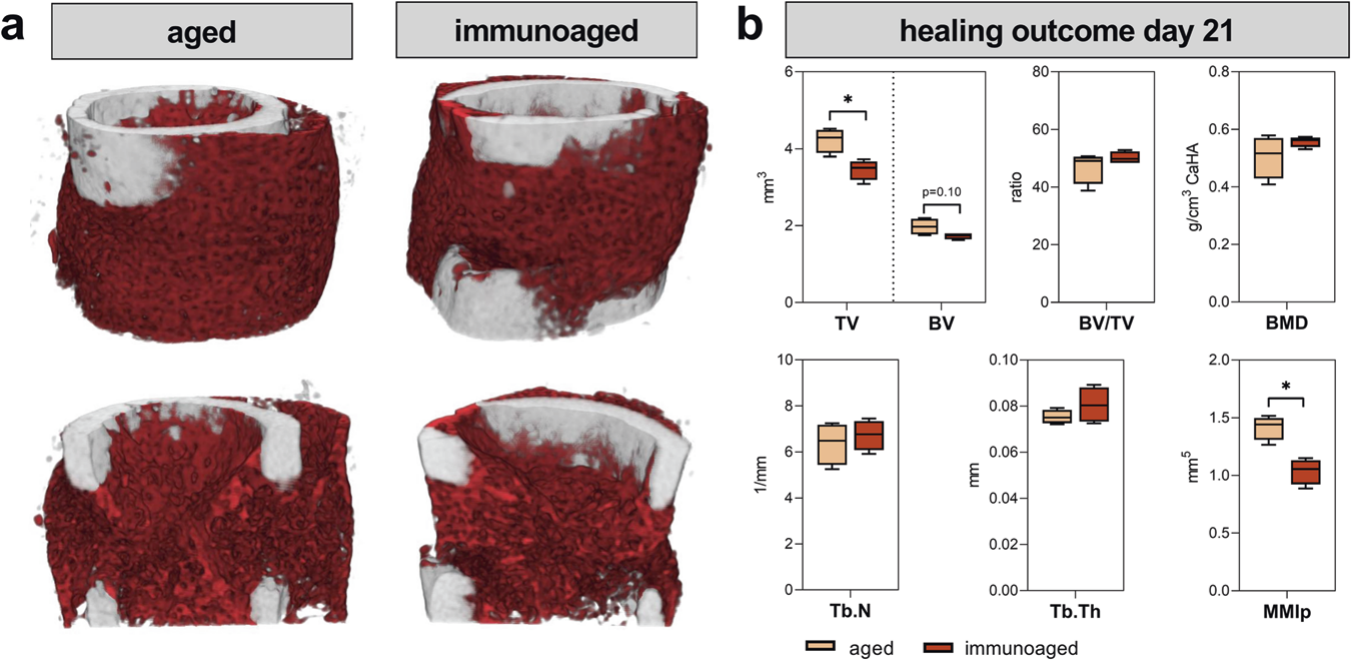
Analysis of the fracture gap after osteotomy in 12-month-old mice revealed a delayed fracture healing process in immunologically experienced (immunoaged) mice. **a** Representative images of the newly formed bone (red) and cortical bone (gray) in the fracture gap 21 days post-surgery, which were analyzed via microcomputed X-ray tomography. **b** Callus volume (TV) was significantly decreased in the immunoaged group, whereas bone volume (BV), bone mineral density (BMD), trabecular number (Tb.N), and trabecular thickness (Tb.Th) were not significantly different between the two groups. The ratio of bone volume to total volume (BV/TV) was not affected by the immunological experience. However, the diminished bone formation in the immunoaged group led to a less stable fracture callus, as shown by a lower minimal polar moment of inertia (MMIp). *N* = 4 per group; box-and-whisker plot with a line at the median; Welch’s *t*-test.

Next, the healing outcomes after long bone fracture was analyzed in 12-month-old mice. An osteotomy was introduced in the left femur of mice with distinct immune experience levels. At 21 days post-surgery, fracture healing was analyzed by microcomputed X-ray tomography (µCT) and revealed a more progressed and more stable callus in the immunologically less-experienced (aged) group compared to a less stable callus tissue in the immunoaged group (Fig. 1a). The callus of the immunoaged group showed a significantly smaller callus volume (TV) than that of the aged group, and the minimal polar moment of inertia (MMIp), a computational analysis of torsional stability, revealed a less stable callus in the immunoaged group (Fig. 1b). The microarchitecture, however, was not significantly altered (trabecular number and thickness), and the mineral density (BMD) of the newly formed bone did not differ between the groups. Adaptive immune experience significantly delayed the fracture healing process, as indicated by a smaller and less stable callus. As the immune system was the only factor that varied between the two groups, systemic and local immunological characteristics, including specific factors driving the immune response and the involvement of progenitor cells, were further analyzed to understand the inferior healing outcome observed in the immunoaged group.

### The microbiome was absent during the initial inflammatory phase and reconstituted at a slower pace with increased immune experience

**Fig. 2:**
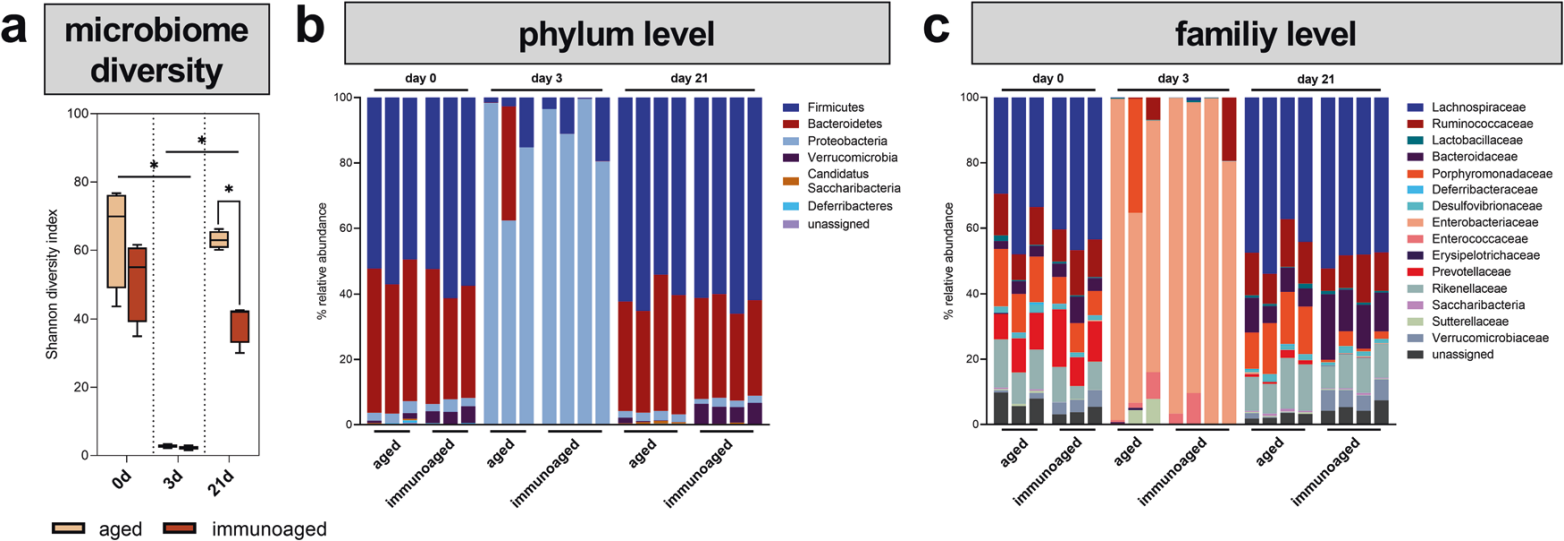
Analysis of the gut microbiome showed substantial alterations post-surgery due to differences in the level of immunological experience. The gut microbiota was analyzed in the cecum and sequenced by 16 S rRNA. **a** The microbiome diversity was significantly decreased post-surgery due to antibiotic treatment at the time of surgery. The reconstitution of the microbiome was significantly slower in the immunoaged group, whereas the aged mice reached presurgery levels 21 days post-surgery. **b** Phylogenic analysis revealed a strong dominance of *Proteobacteria* 3 days post-surgery. **c** Bacterial families significantly differed between immunoaged and aged mice pre-surgery as well as 21 days post-surgery. *N* = 3-4 per group; box-and-whisker plot with a line at the median and bar plots; Welch’s *t*-test.

The microbiome was recently reported to steer inflammatory processes and train the immune system^35^. To shed light on the confounding factor of the microbiome, the microbiome was analyzed in the cecum during fracture healing. The less-experienced (aged) and immunoaged groups were analyzed at a presurgery timepoint, during the inflammatory phase (3 days post-surgery), and after bone consolidation (21 days post-surgery). Presurgically, the microbiome diversity did not differ significantly between the aged and immunoaged groups, although there was a trend toward less diversity in the latter group. In both groups, the microbiome was almost absent 3 days post-surgery due to short-term antibiotic treatment of the animals. Thereafter, the microbiome was reconstituted at different speeds and was slower in the immunoaged group than in the aged group at 21 days post-surgery (Shannon diversity index, Fig. 2a). The microbiome phyla and family analyses showed differences between the two groups. Clindamycin, the antibiotic used in this study, shifted the community composition to a predominance of *Proteobacteria* belonging to the family *Enterobacteriaceae*. Three days post-surgery, most of the remaining bacteria were classified as *Proteobacteria*, a major phylum of Gram-negative bacteria and the dominant bacteria in the gastrointestinal tract, whereas in the presurgery group, *Firmicutes* and *Bacteroidetes* represented the dominant phyla (Fig. 2b). The microbiome family analysis showed a rich population diversity in this preclinical model presurgically and 21 days postsurgically, while a significant decrease in diversity was observed 3 days post-surgery.

Interestingly, under the influence of the experienced adaptive immune system, *Porphyromonadacae* showed less effective reconstitution 21 days post-surgery, whereas *Verrucomicrobiacacae* showed a more pronounced repopulation of the gastrointestinal tract (Fig. 2c). Within both groups, *Prevotellaceae* also showed a disturbed reconstitution ability 21 days post-surgery. With immunological experience, the gut microbiota showed a significant delay in returning to the baseline state. Considering our focus on the early inflammatory events during fracture healing, the microbiome could be excluded as an influencing factor of immunologically driven delayed healing.

### Local immune contribution of the T-cell compartments in aged mice compared to immunoaged mice after injury

**Fig. 3:**
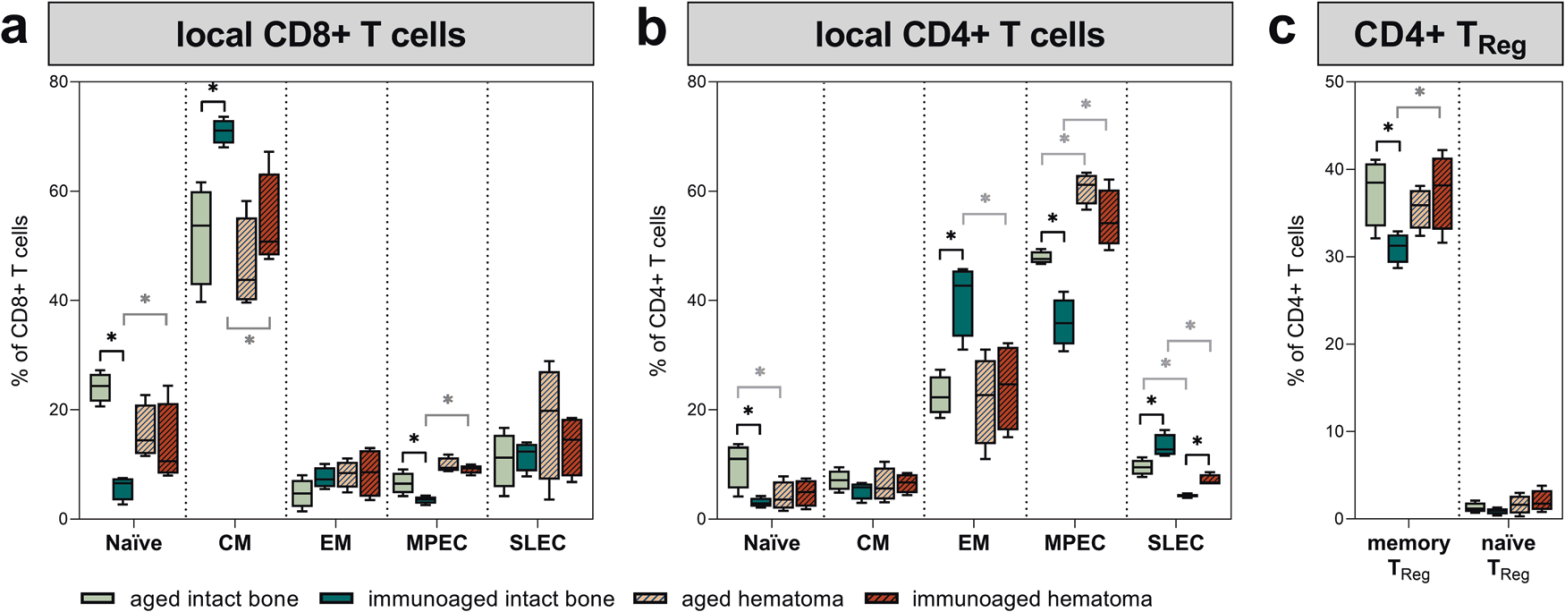
Immune phenotype analysis of local T cells at the site of injury in comparison to the bone marrow from untreated mice 3 days post-surgery revealed significant differences in T-cell subtypes. The fracture gap, including the hematoma and the adjacent bone marrow, was analyzed by flow cytometry 3 days post-surgery and compared to untreated bone marrow from the same groups. Subpopulations of CD8+ (**a**) and CD4+ (**b**) T cells were analyzed. Initially, memory formation was more advanced in immunoaged mice, as indicated by increases in memory populations and decreases in naïve T-cell numbers. However, these differences were equalized 3 days post-surgery at the fracture site. Significant differences at 3 days post-surgery could only be observed in short-lived effector cells (SLECs) in the CD4+ T-cell compartment around the fracture site. The osteotomy significantly changed the memory populations in CD4+ T cells: The percentage of effector memory (EM) T cells was diminished, whereas the percentage of memory precursor cells (MPEC) were significantly increased compared to that in the untreated bone marrow. **c** CD4+ regulatory T-cell numbers were increased in the immunoaged group after injury, and these cells were also less abundant in the untreated bone marrow of untreated mice. *N* = *4* per group; box-and-whisker plot with a line at the median; Welch’s *t*-test; black * = between immunological experience groups, gray * = between untreated and osteotomized groups.

To identify key immune cell signaling pathways causing the divergent healing outcomes observed after bone injury, the local contributions of immune cells during the initial inflammatory response after injury were monitored in fracture hematoma. The fracture gap, including the hematoma and the adjacent bone marrow, was analyzed by flow cytometry three days post-surgery. The hematoma consists of locally available immune cells as well as attracted immune cells from the surrounding tissue and blood vessels. Here, we analyzed the local immune phenotype of T cells in the bone marrow from untreated mice in comparison with the hematoma phase in osteotomized mice. Immunoaged mice significantly differed in terms of the immune cell composition of the bone marrow from intact bones compared to that of untreated aged mice with less immunological experience. In the intact bone marrow, fewer naïve CD8+ T cells were observed, and they were superseded by memory cells, with a significantly increased level of central memory CD8+ T cells in the immunoaged group (Fig. 3a). In the CD4+ T-cell compartment, a similar distribution could be observed; however, effector memory T cells were predominant. In particular, the level of effector cells differed between the experience levels, as fewer MPECs could be observed in the immunoaged group, but SLECs were more abundant (Fig. 3b). The level of regulatory CD4+ T cells was significantly higher in the less-experienced (aged) group, showing a more pronounced attenuation and regulation of the adaptive immune system (Fig. 3c). The osteotomy severely altered the local composition of immune cells: The aged group aligned to some extent with the immunoaged group with regard to fewer naïve cells locally at the fracture site. The immunoaged group, however, showed a significantly increased local contribution of naïve CD8+ T cells (Fig. 3a). In both groups, more MPECs were present at the fracture site than in the intact bone of the untreated mice. The CD4+ T_Reg_ population increased simultaneously in the immunoaged group. The CD4+ MPEC population increased in both groups, showing an active contribution of effector cells during fracture healing. An interesting finding revealed that the CD4+ T_EM_ cells reached similar levels in both groups on day 3 post-surgery. Significant alterations in the adaptive immune cell phenotype could consequently be observed at the injury site during the initial inflammatory phase, revealing a tight interaction between adaptive immune cells and fracture healing. In all groups, the analyses revealed the dynamic changes that are initiated in the bone marrow after osteotomy.

### Immunological experience leads to stronger pro-inflammatory responses via more pronounced activation patterns and the absence of CXCR3 downregulation

**Fig. 4:**
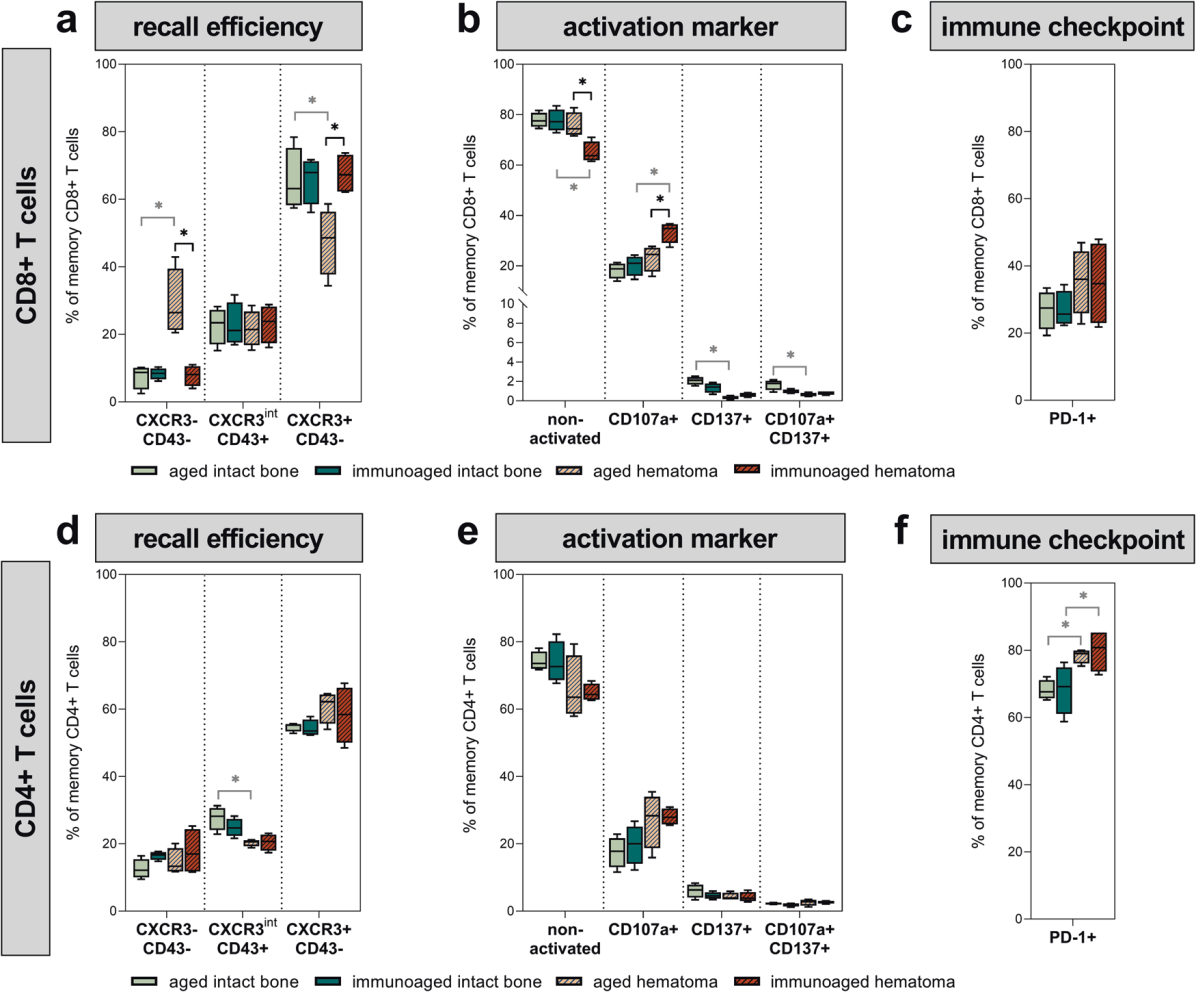
Analysis of the local T cells at the site of injury 3 days post-surgery in comparison to the bone marrow from untreated mice revealed significant differences in T-cell activation states. **d**, **f** Introducing a fracture decreased CXCR3 presentation on the cell surface in CD4+ T cells and increased the expression of PD-1 accordingly. **a** Interestingly, CXCR3 expression on CD8+ T cells was highly regulated by the initial experience level, as in aged mice, CXCR3 expression on CD8+ T cells 3 days post-surgery was downregulated, which was not seen in immunoaged mice. **c** However, there was no significant increase in PD-1 surface expression on memory and effector CD8+ T cells, and less PD-1 expression on memory/effector T cells resulted in escape from immune regulation. **b**, **e** Introducing a fracture activated CD8+ memory T cells, as shown by increased levels of CD107a on the cell surface. CD8+ T cells showed a significantly higher activation state in immunoaged mice 3 days post-surgery than in the aged-only group. *N* = 4 per group; box-and-whisker plot with a line at the median; Welch’s *t*-test; black * = between immunological experience groups, gray * = between untreated and osteotomized groups.

Various subtypes of memory T cells (CD44+ CD62L±) were found to adjust to comparable levels locally at the fracture site for the groups with different immunological experience levels. Analyzing the activation and inhibition patterns within the fracture hematoma samples, however, revealed substantial differences between the groups. The chemokine receptor C-X-C motif chemokine receptor 3 (CXCR3) and the adhesion molecule sialophorin (CD43) are important surface proteins on memory T cells, indicating their efficiency in inducing a recall response. CXCR3-CD43- memory T cells show a low secretion of effector cytokines upon activation, whereas CXCR3+ CD43- memory T cells are prone to massive effector cytokine secretion, and CXCR3int CD43+ memory T cells have an intermediate effector function^36^. Local CD8+ memory T cells in the fracture gap showed significant downregulation of CXCR3 expression on the cell surface in the less-experienced (aged) group. For CD4+ memory T cells, both groups showed a decrease in the intermediate (CXCR3int CD43+) effector function (Fig. 4a, d). CD8+ memory T cells in the immunoaged group showed a more activated phenotype due to a lack of CXCR3 downregulation and upregulation of the activation marker CD107a (upregulated upon cytokine secretion and cytotoxic activity). CD137, a marker for antigen-specific activation, however, was downregulated following osteotomy, but no difference between the groups was observed (Fig. 4b). CD107a expression generally correlates with cytokine release, while the expression of CXCR3 and CD43 correlates with the level and intensity of effector cytokines, indicating an overall more pronounced inflammatory phenotype of CD8+ memory/effector T cells in the immunoaged group. A slight increase in the inhibitory marker PD-1 on CD8+ memory T cells was observed in both groups during the initial hematoma phase 3 days post-surgery (Fig. 4c). CD4+ memory T cells showed no significant difference in activation marker expression, but both groups showed a significant increase in PD-1 expression on the cell surface as an indicator of activation (Fig. 4e, f). These findings show that the two different levels of adaptive immune experience within the fracture hematoma are associated with similar immune cell compositions. Nevertheless, distinct differences in cell activation and pro-inflammatory abilities were observed across hematomas in the early healing phase, with pronounced pro-inflammatory signaling potential in the immunoaged group.

While adaptive immunity displayed distinct differences in the hematoma from early on, it may be speculated that innate immunity, which is tightly coupled to adaptive immunity, may also show crosstalk during immunological response to injury. Thus, we further analyzed cells of the innate immune system locally at the osteotomy site.

### Innate immune cells accumulating at the fracture site help to maintain a prolonged inflammatory phase under immunoaged conditions

**Fig. 5:**
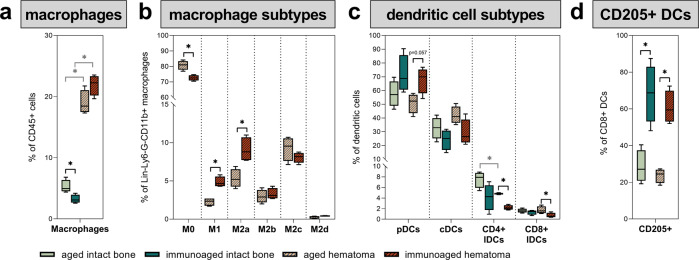
Analysis of the local macrophage and dendritic cell populations at the site of injury 3 days post-surgery revealed significant differences in macrophage polarization, activation, and dendritic cell subsets. Macrophages and dendritic cells were analyzed via flow cytometry in the untreated bone marrow and at the fracture site in the immunoaged and aged groups. **a** Three days post-surgery, significantly more macrophages were present around the fracture gap than in the untreated bone marrow. **b** The activation state of macrophages at the fracture site differed, as significantly more M1-polarized macrophages could be found in the immunoaged group than in the aged group 3 days post-surgery. Additionally, an increased level of M2a-activated macrophages was observed in the immunoaged group. These data showed an increased activation state in the mice with higher adaptive immune experience and depicted a prolonged inflammatory phase. **c** Immunoaged mice showed an increased level of plasmacytoid dendritic cells (pDCs) at the fracture site compared to that in aged mice 3 days post-surgery. In contrast, increased levels of specified CD4+ and CD8+ lymphoid tissue-resident classical dendritic cells (lDCs) could be found in aged mice compared to immunoaged mice. **d** Increased adaptive immune experience significantly altered the innate immune system by increasing CD205+ expression on CD8+ lDCs. Higher expression levels of CD205+ on DCs facilitate communication with CD8+ T cells. *N* = 4 per group; box-and-whisker plot with a line at the median; Welch’s *t*-test; black * = between immunological experience groups, gray * = between untreated and osteotomized groups.

Macrophages are key factors in the healing process. They are readily available after injury and are among the first cells arriving at the fracture site. Macrophages are essential for successful healing. The osteotomy almost quadrupled the proportion of the macrophage population in the hematoma, making them approximately 20% of all leukocytes (Fig. 5a). Analysis of the macrophage subtypes in the hematoma revealed that macrophages in the immunoaged group showed a prolonged activation phenotype. Macrophages can be differentiated into M0 or different polarized states, namely, M1 (pro-inflammatory state) and M2 macrophages (alternatively activated state). M2 macrophages can be further subdivided into different activation subtypes, namely, M2a, M2b, M2c, and M2d macrophages, which are all attributed to distinct cytokine secretion profiles and functions. In the immunoaged group, more macrophages were activated, with fewer M0 (inactive macrophages) remaining than in the aged group. Additionally, compared to the aged group, the immunoaged group showed significantly more M1-activated macrophages, which are known for their pro-inflammatory properties. M2-polarized macrophages showed differences in their activation states, and M2a macrophages were more abundant at the fracture site in the immunoaged group than in the aged group 3 days post-surgery (Fig. 5b). Overall, the increased polarization and activation state of macrophages in the immunoaged group indicate an increased inflammatory state.

In addition to the macrophage population, dendritic cells contribute to the inflammatory state via the cross-presentation of molecules and are known for their direct stimulation and modulation of CD8+ T cells. CD205+ CD8+ dendritic cells in particular directly guide and communicate with CD8+ T cells in mice. Different DC subsets exist in mice, with distinct functions and cytokine secretion patterns. Here, we monitored three distinct DC populations, namely, plasmacytoid dendritic cells (pDCs), conventional DCs (cDCs), and lymphoid tissue-resident classical DCs (lDCs). Similar to the macrophage subtypes, the dendritic cell subtypes were affected by the experience of the adaptive immune system: More plasmacytoid dendritic cells (pDCs) and fewer conventional DCs (cDCs) and CD4+ lymphoid tissue-resident classical DCs (lDCs) could be identified in the hematoma in the immunoaged group compared to the aged group. The population of CD8+ lDCs was rather small, and significantly smaller percentages were detected in the immunoaged group (Fig. 5c). However, in the immunoaged group, more than 60% of all CD8+ DCs were CD205+, indicating close communication between innate and adaptive immune cells, as only ~25% of cells were CD205+ in the aged group (Fig. 5d). CD8+ DCs in mice usually start a type I cytotoxic immune response and are known to be involved in CD8+ T-cell activation, including antigen cross-presentation and cytokine production. Hence, these cells play a major role in the induction of CD8+ T-cell immune responses. DCs play a critical role in immunomodulation by balancing tolerance and immune responses and are able to orchestrate the adaptive immune response.

Overall, the cells of the adaptive and innate immune responses jointly orchestrate and thereby steer the inflammatory cascade. However, stromal progenitor cells are key for laying down the matrix and thus restoring the injured tissue by producing and remodeling the extracellular matrix. Hence, we focused on the stromal progenitor cells involved in these early stages of fracture healing in the next step.

### Subtypes of mesenchymal stromal cells adapt to the local adaptive immune experience in the hematoma

**Fig. 6:**
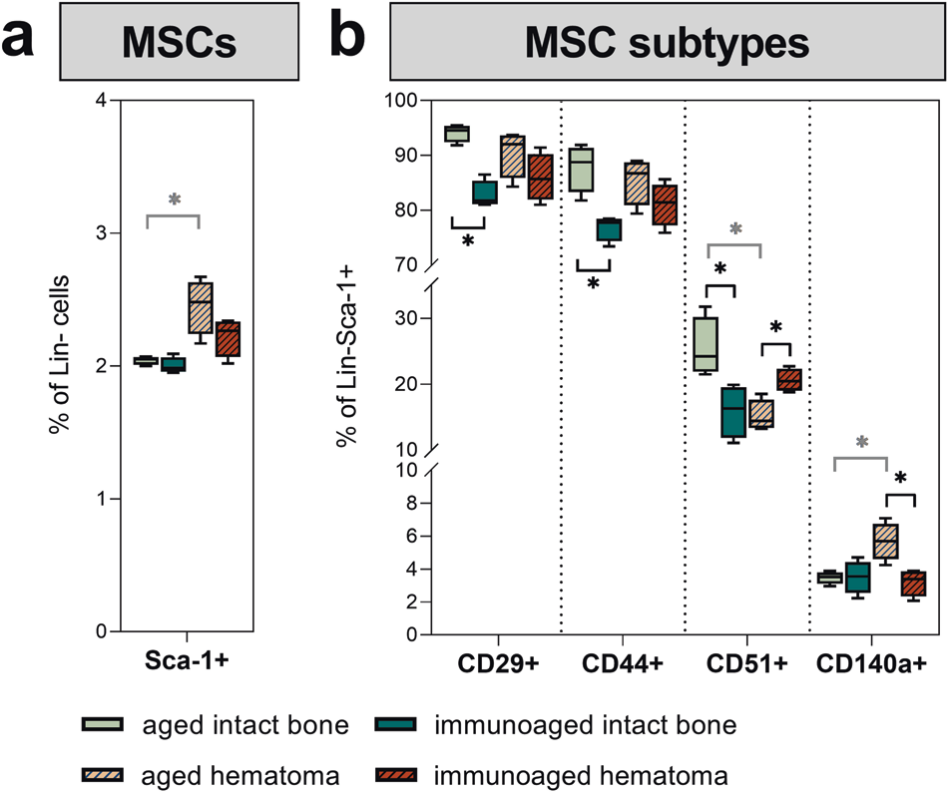
Analysis of local mesenchymal stromal cell subtypes at the site of injury 3 days post-surgery revealed significant differences in subtype distribution and priming. Mesenchymal stromal cells (MSCs) were analyzed via flow cytometry in the untreated bone marrow and at the fracture site in the immunoaged and aged groups. **a** Flow cytometric analysis of bone marrow from the untreated bone versus the fracture hematoma three days post-surgery. Osteotomy increased the availability of MSCs (CD45-CD34-CD31-(Lin)Sca-1+) at the fracture site. The mobilization and recruitment of MSCs was significantly increased in the aged group. The immunoaged group, however, showed less proliferation and migration. **b** The heterogeneity of MSC subtypes also differed between groups with different immunological experience levels. Specialized MSCs were present at different frequencies, as a higher percentage of MSCs in the aged mice were PαS cells (Sca-1+ CD140a+), whereas a higher percentage were murine skeletal stem cells (mSSC, Sca-1+ CD51+) in the immunoaged mice. *N* = 4 per group; box-and-whisker plot with a line at the median; Welch’s *t*-test; black * = between immunological experience groups, gray * = between untreated and osteotomized groups.

Mesenchymal stromal cells (MSCs) are considered key factors in launching the regeneration process itself. Not only can they differentiate into chondroblasts or osteoblasts, but they are also known for their potent immunomodulatory capacity. Increasing the experience of adaptive immune cells changed the crosstalk between immune cells and MSCs and altered cellular phenotypes in the MSC pool. In the hematoma, the number of pure MSCs (CD45-CD34-CD31-Sca-1+) increased on day 3 post-surgery. In the less-experienced (aged) group, this increase was statistically significant compared to the intact bones. However, this increase in MSC numbers was smaller in the immunoaged group, illustrating slower mobilization or recruitment to the osteotomy site (Fig. 6a). The MSC composition diverged between the two groups with distinct immunological experience levels when compared with the intact bone marrow. Significantly fewer MSCs expressed CD29 and CD44, and fewer murine skeletal stem cells (mSSC, CD51+) were found in the immunoaged group than in the aged group. Three days after osteotomy, however, the immunoaged group showed more mSSCs (CD51+), whereas the aged group showed a higher mobilization and recruitment of PαS cells (PDGFRα (CD140a) and Sca-1 expressing cells) to the fracture site. The numbers of PαS cells did not differ between the osteotomy site and intact bone marrow in the immunoaged group (Fig. 6b). Analyzing MSC subtypes along with immune cells showed a compensatory effect on the healing process due to the increased immunological experience in the immunoaged group. The experience level impacted the composition of MSC subtypes, indicating an altered interaction between immune cells and stromal progenitor cells. To analyze eventual changes in cellular crosstalk via cytokines, we analyzed the diverging cytokine patterns induced by distinct immunological experience levels.

### Cytokine levels depict significant alterations in the communication pattern between immune and mesenchymal precursor cells in the immunoaged group

**Fig. 7:**
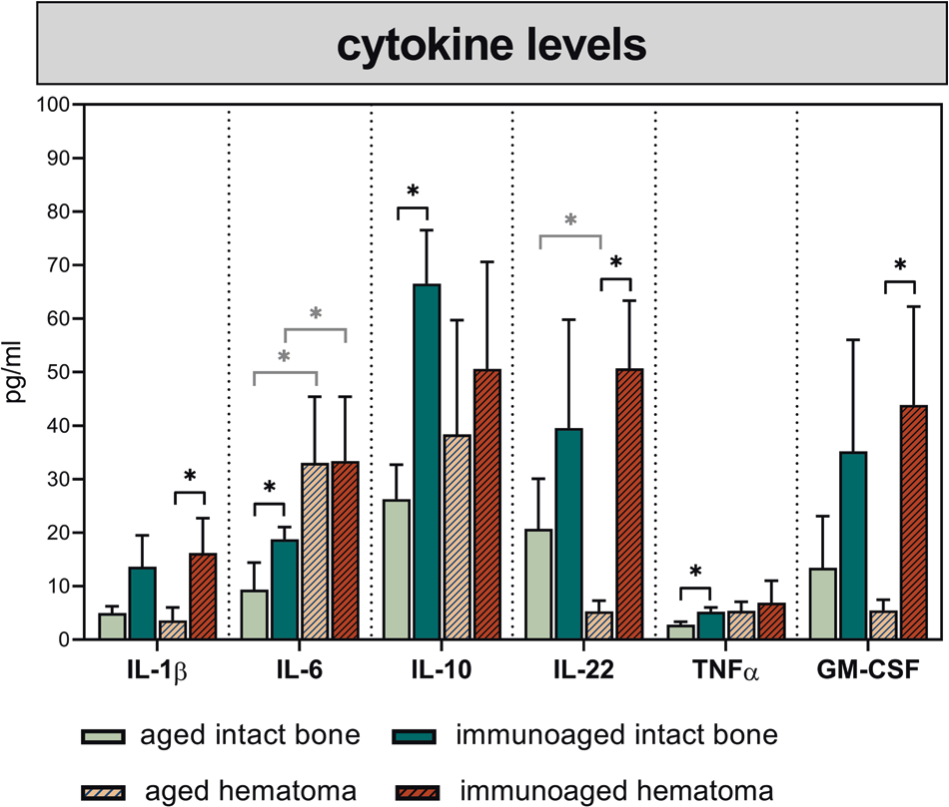
Cytokine patterns in the basal state and in response to an injury showed increased reactivity in immunoaged mice. Systemic cytokine levels in the blood were screened pre-surgery and 3 days post-surgery for immunoaged and less-experienced (aged) 12-month-old mice. The cytokine levels in the basal state revealed an inflammaged status in immunoaged mice, as indicated by increased systemic levels of TNFα, IL-6, and IL-10. Upon activation due to osteotomy, the cytokine levels varied based on the immune experience level: Increased levels of IL-22, GM-CSF, and IL-1b could be observed in immunoaged mice. Most importantly, IL-22 not only showed high basal expression levels in immunoaged compared to aged mice but also appeared to react distinctly in the respective immunoaged phenotype, with a significant downregulation upon injury in aged mice but an increase upon injury in immunoaged mice. *N* = 4 per group; bar plot with SD; Welch’s *t*-test; black * = between immunological experience groups, gray * = between untreated and osteotomized groups.

Immune cell communication with adjacent cells is mostly performed in a paracrine manner by secreting cytokines. To analyze the altered communication of experienced immune cells in vivo during the fracture healing process, the secreted cytokines were screened via a multiplexed immunosorbent assay (Multiplex ELISA). The systemic cytokine levels were measured in animals from the same aged and immunoaged groups as above, comparing presurgery (untreated mice), 3 days post-surgery, and 21 days post-surgery. For a complete list of cytokines/chemokines and timepoints analyzed, please refer to the supplementary material (Supplementary Figs. 2,3). The most striking differences measured at the presurgery timepoint and 3 days post-surgery are shown in Fig. 7. The tumor necrosis factor α (TNFα) level was found to be systemically increased in 12-month-old untreated mice with an experienced immune system (immunoaged group) compared to mice of the same age but with a less-experienced immune system (aged group), illustrating the inflammaged status of these mice by a systemic low-grade pro-inflammatory milieu. Interestingly, interleukin (IL)-10 levels were likewise increased in the baseline state in the immunoaged group, a typical sign of counter-regulation. Three days post-surgery, during the inflammatory phase of fracture healing, significantly increased levels of cytokines (IL-1b, IL-22, and granulocyte-macrophage colony-stimulating factor (GM-CSF)) could be observed in the immunoaged group compared to the aged group. While IL-6 seemed to be generally induced by the osteotomy itself (in both groups), IL-22 was significantly downregulated in the aged group compared to the intact group but significantly upregulated in the immunoaged group. IL-22 is secreted by immune cells from both the innate and adaptive immune systems, but the major source is memory/effector Th22 cells. Interestingly, the IL-22 receptor can be found only on nonhematopoietic cells, such as endothelial, epithelial and other stromal cells. GM-CSF can be secreted by varyious immune cell populations as well as by endothelial and other stromal cells, such as fibroblasts. GM-CSF can induce the proliferation of macrophages but can also polarize macrophages toward the M1 lineage. Higher GM-CSF levels were detected and consistent with the increased proportion of M1 macrophages at the osteotomy site in the immunoaged group (see Fig. 5b).

### Immune cell communication via IL-22 and GM-CSF drives differentiation, migration, and proliferation in vitro

The cytokine milieu significantly diverged between the experience levels in both 12-month-old mouse groups. IL-22 and GM-CSF were selected as potential cytokines that directly interfere with cells during early regeneration at the onset of healing. Both cytokines were tested in vitro for their impacts on key regenerative processes, such as osteogenic, adipogenic, and chondrogenic differentiation of MSCs, as well as their migratory and proliferative potential (scratch assay). Finally, the effect of these cytokines on endothelial cell tube formation was analyzed. For osteogenic and adipogenic differentiation, the cytokines were tested at three different concentrations, low (1 ng/ml), medium (10 ng/ml), and high (100 ng/ml). For the other assays, the concentrations were adapted according to preliminary experiments. To increase the translatability of findings obtained with this preclinical animal model, the effect of IL-22 and GM-CSF on stromal and endothelial progenitor cells was analyzed with human recombinant proteins in human primary cells. Furthermore, the gene sequence and structure of IL-22 and GM-CSF are highly conserved between humans and mice.

**Fig. 8:**
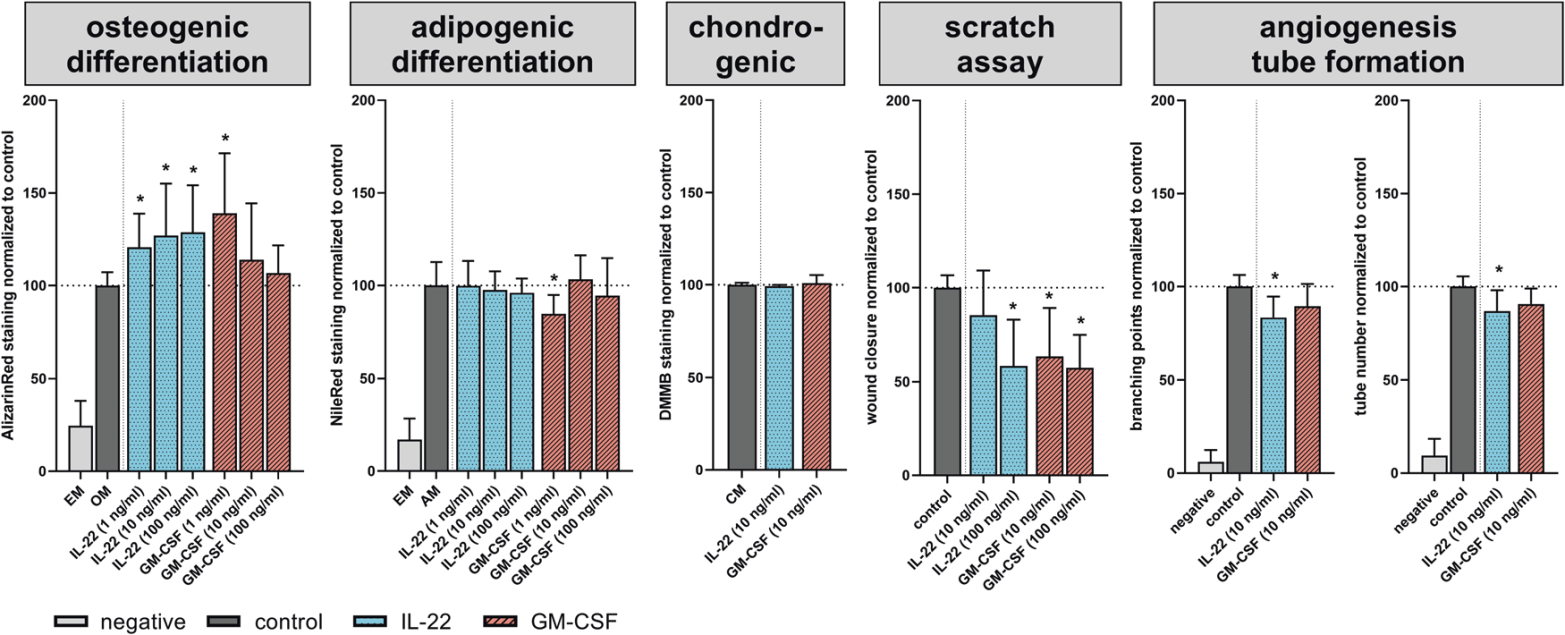
IL-22 and GM-CSF interfere with the regenerative potential of progenitor cells. The two cytokines were tested individually at different concentrations to determine their effects on the differentiation and migratory/proliferative potential of mesenchymal stromal cells as well as the angiogenic potential of endothelial cells to form new tubes in vitro. IL-22 increased the mineralization of the extracellular matrix in the osteogenic differentiation assays, but higher concentrations of IL-22 decreased migratory and proliferative potential and inhibited the de novo formation of new vessels by reducing the tube number and branching points. GM-CSF predominantly interfered with the migratory and proliferative behavior of MSCs but did not inhibit the angiogenic potential of endothelial cells. EM expansion medium, OM osteogenic medium, AM adipogenic medium, CM chondrogenic medium; *n* = 5 MSC donors per assay, *n* = 5 pooled endothelial cell (HUVEC) donors, tested in triplicate; unpaired *t*-test.

Interestingly, both cytokines induced mineralization of the extracellular matrix (ECM) of MSCs in osteogenic differentiation assays at low concentrations. However, this inductive effect was absent at higher GM-CSF concentrations. Adipogenic differentiation and lipid storage in the cytoplasm were not affected by either cytokine, except for GM-CSF at low concentrations. Under the influence of GM-CSF at a low concentration (1 ng/ml), significantly fewer fat vacuoles could be observed compared to the untreated control cells. The chondrogenic differentiation process was not affected by IL-22 or GM-CSF at osteoinductive concentrations. However, the migratory capacity of MSCs was significantly reduced under the influence of both cytokines, except for IL-22 at moderate (10 ng/ml) concentrations. In the scratch assay, significantly more time was needed to close the gap than observed in the untreated control. In addition to migration, endothelial cell tube formation was significantly blocked by only IL-22. IL-22 significantly reduced the ability to form new tubes by decreasing branching between neighboring tubes (branching points), and significantly fewer new tubes were formed (Fig. 8 and Supplementary Fig. 4). These in vitro experiments indicate that the cytokines released by immune cells in the immunoaged group directly interfered with regenerative processes related to MSCs and endothelial cells.

### IL-22 is a central immune cytokine that modulates inflammatory cascades during regeneration in vivo

IL-22 was shown to interfere with all major processes that are considered to be essential for regeneration during bone healing: proliferation and migration of progenitor cells, osteogenic differentiation, and angiogenesis. To investigate whether this immune cell signal is of special importance to the regenerative process overall, an in vivo proof-of-concept experiment was conducted in which IL-22 was supplemented in an aged group (expecting deteriorating bone healing) or neutralized in an immunoaged group (expecting enhanced bone healing) of 12-month-old mice.

**Fig. 9:**
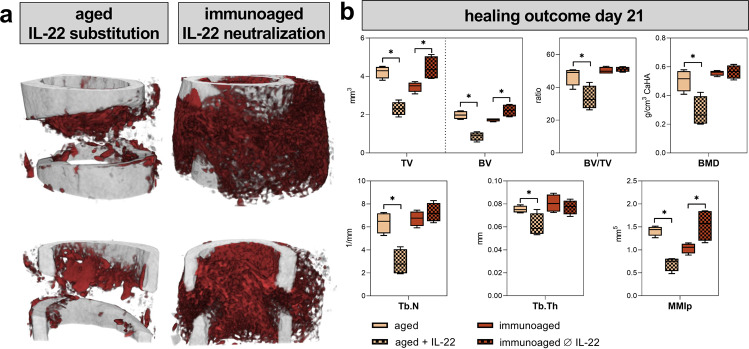
Substitution of IL-22 in less-experienced (aged) mice impaired the fracture healing process, whereas neutralization of IL-22 accelerated or ameliorated the healing process in experienced (immunoaged) mice. An osteotomy was introduced in the left femur, the endogenous IL-22 levels were altered via neutralization with antibodies or supplementation with recombinant IL-22 protein, and the healing outcome was assessed 21 days post-surgery with µCT. **a**, **b** In less-experienced (aged) 12-month-old mice, which had a lower level of IL-22 3 days post-surgery, IL-22 was supplemented during the first 7 days post-surgery, starting 24 h post-surgery. Fracture healing was almost completely abolished 21 days post-surgery under IL-22 supplementation. The callus volume (TV) and the bone volume (BV) were significantly decreased, and the microarchitecture (trabecular number Tb.N and trabecular thickness Tb.Th) and bone mineral density (BMD) significantly deteriorated. Immunoaged 12-month-old mice showed a higher level of IL-22 3 days post-surgery, and IL-22 was neutralized during the first 7 days post-surgery, starting 24 h post-surgery. Neutralization of IL-22 boosted the bone healing process, as an increased callus volume and bone volume could be observed 21 days post-surgery. The stability of the fracture gap was significantly increased (MMIp), and callus volume and stability reached the levels observed in less-experienced (aged) mice. *N* = 4–6 per group; box-and-whisker plot with a line at the median; Welch’s *t*-test.

In the aged group, which had decreased levels of IL-22 3 days post-surgery, recombinant IL-22 was supplemented during the initial 7 days post-surgery. In the immunoaged group, which showed elevated IL-22 levels, IL-22 was neutralized via an IL-22 neutralizing antibody to allow the contrary regulation of endogenous IL-22. The healing outcomes were analyzed at 21 days post-surgery, during the hard callus phase. In both groups, IL-22 treatment showed significant effects. The less-experienced (aged) 12-month-old group receiving recombinant IL-22 showed significantly inferior healing, as indicated by decreased callus volume (TV) and decreased newly formed bone volume (BV). In contrast, when IL-22 was neutralized in the immunoaged group, healing progression was rescued and showed qualities comparable to those observed in the aged group. TV and BV were significantly increased after IL-22 neutralization when compared to the immunoaged healing group without intervention. Additionally, the minimal polar moment of inertia (MMIp), an indicator of bone torsional stability, showed increased values for the callus 21 days post-surgery after immune modulation (Fig. 9a, b). Neutralizing IL-22 during the healing process in immunologically experienced mice induced an accelerated healing process and a healing outcome similar to that seen in the aged group. Supplementation of IL-22, on the other hand, nearly abolished the healing process in the aged group with an otherwise more naïve, more potent immune composition.

## Discussion

The variations in healing outcomes in aged individuals may be linked to specific alterations in cellular phenotype and functionality in immunoaged individuals due to distinct cytokine patterns. Herein, we reveal for the first time the central role of IL-22, which in high abundance was associated with a diminished healing capability in aged individuals. IL-22 mediates both protective and pathogenic effects on various tissue cells depending on timing, environmental factors, and concentration. Increased adaptive immune experience] intensified and prolonged the initial inflammatory phase after injury, coinciding with high expression of IL-22 and hampered regeneration. Not only in response to injury but also during homeostasis, an increased adaptive immune experience reflected an elevated inflammatory status, termed immunoaging/inflammaging. Consequently, patients with an increased immunological experience, more frequent among elderly individuals, have an increased probability of suffering from delayed healing^15^.

We and others showed the detrimental effects of different immune cell types during fracture healing^37–39^. In the present study, we provide evidence that increased adaptive immune experience directly affected the healing process after a fracture. Exposure of mice to environmental microorganisms by reducing ventilation shielding significantly promoted the formation of effector and memory cells of the adaptive immune system. In comparison to mice housed under specific pathogen-free (SPF) conditions, this preclinical model allows us to discriminate the influence of immune experience and aging per se on regeneration. Previous studies have revealed that aged mice exhibit a reduced capacity for regeneration^25,40–42^. However, this study identified the specific impact of immune experience in aged individuals on mineralization, biomechanical stability, systemic and local innate and adaptive immune cell composition, the microbiome, and MSC subset composition during homeostasis and regeneration. By including a comprehensive analysis of those factors currently considered to influence bone healing, we were able to show that immunological experience is a determining factor in healing success and thus a possible therapeutic target to counter the age-induced decrease in regenerative capacity.

Immunoaging achieved by exposing the mice to environmental microorganisms altered the microbial diversity in aged animals. The microbiome in immunoaged mice is comparable to that in wild mice^43^. However, the use of antibiotics during surgery significantly reduced the potential effect of the microbiome, and the interaction with the immune system, as indicated by the effective diversity index, was found to be nearly zero after antibiotic treatment. The microbiome was significantly influenced by immunological experience during reconstitution after antibiotic treatment. Future studies are needed to unravel the distinct interactions of immunoaging and microbial diversity and the respective impacts on regenerative processes.

This study showed an amplified and prolonged immune reaction of the adaptive immune system, especially CD8+ T cells, locally at the fracture site in immunologically experienced (immunoaged) mice compared to less-experienced aged mice. This prolonged and amplified pro-inflammatory reaction was due to upregulated activation markers, increased chemokine receptor availability, and a lack of inhibitory signaling. These alterations in the phenotype and functionality of specific immune cell types might be the underlying cause of subsequent delayed healing in immunologically experienced mice. Analyzing the local inflammatory response after injury revealed amplification and prolongation of the initial inflammatory phase under immunoaged conditions. Increased levels of CXCR3 on the surface of CD8+ T cells were found to correlate with increased effector functions in CD8+ T cells in other studies and were associated with disease severity, supporting our findings^36,44–46^. High levels of CXCR3 also facilitate the migratory behavior of CD8+ T cells^47^. Less-experienced mice showed a decreased level of CXCR3 on CD8+ T cells during the initial inflammatory process during bone healing, revealed less invasive behavior and diminished effector function. As late-stage effector CD8+ T cells could be attributed to delay the healing after injury, timely and local downregulation of CXCR3 (as seen in our aged, more naïve group; Fig. 4) favors the progression of healing. Further studies are needed to reveal the contribution of CXCR3 during fracture healing and the time course of regulation of this chemokine receptor.

Markers that indicate an active state of T cells are CD107a and CD137, with CD107a being considered a nonspecific activation marker, whereas CD137 is an activation marker indicating the involvement of the T-cell receptor (TCR)^48,49^. Immunoaged mice showed increased levels of CD107a compared to those of the less-experienced (aged) mice during the initial inflammatory phase. Enhanced active states of CD8+ T cells result in higher expression of pro-inflammatory cytokines at the osteotomy site in the immunoaged group. PD-1, however, limits the effector function of T cells in pathogenic situations (e.g., as found in cancer^50,51^) and appears to limit the activity of T cells. PD-1 upregulation on T cells was also found in fracture healing, as shown in the present study. This marker with inhibitory signaling effects on T cells was upregulated three days post-surgery on CD4+ T cells, while a related upregulation of PD-1 on CD8+ T cells was not observed (Fig. 4).

The innate immune system is a prerequisite for successful healing, and depletion of macrophages^52,53^, and reduction of neutrophil granulocytes^54^ or deficient mast cells^55^ hampers or even abolish the fracture healing process. Macrophages show high plasticity of effector functions and function as potent regulators of the regenerative process by initiating and steering the inflammatory cascade. This study comprehensively analyzed macrophage plasticity locally at the fracture site by not only discriminating M1- and M2-polarized macrophages but also differentiating the activated M2 subtypes, namely, M2a, M2b, M2c, and M2d. All subtypes show distinct differences in their secretomes and perform diverse functions: M2a is known as the classically alternatively activated macrophage subtype, with a known anti-inflammatory secretome, such as IL-10 and TGFβ. M2b macrophages, also known as type 2, are producers of IL-1, IL-6, IL-10, and TNFα. M2c macrophages are described as a more deactivated phenotype, with a secretome comparable to that of M2a macrophages, and M2d macrophages are producers of vascular endothelial growth factor (VEGF) and known for proangiogenic effector functions^56,57^. Immunoaged mice showed an alternative activation state of macrophages locally at the fracture site. M1 and M2a macrophage frequencies were increased, and the cells exhibited a more pronounced inflammatory state (pro- and anti-inflammatory) in the immunoaged group compared to the aged group. Due to the limitation of the timely resolution, it remains to be clarified whether the increased levels of M2a macrophages in the immunologically experienced group are a response to counteract the M1 macrophages or if these two activated macrophage subtypes are independently regulated. Nevertheless, increased numbers of activated macrophages (M1 and M2a) might result in increased cytokine levels and therefore increased cellular stress. The increased levels of GM-CSF observed in the immunoaged group could also contribute to increased M1 activation, as GM-CSF is the main driver of M1 polarization. Endogenous levels of intracellular reactive oxygen species (ROS) were slightly increased locally at the fracture site in the immunoaged group (see Supplementary Fig. 1).

Dendritic cells are key factors in signal transmission to T cells, especially CD8+ CD205+ dendritic cells, which are known to interact closely with CD8+ T cells and CD4+ T cells. The CD205+ dendritic cell population is responsible for the cross-presentation of apoptotic cell-derived antigens^58^. Osteotomy significantly increased CD205 expression on CD8+ DC numbers locally at the fracture site, as shown in this study.

As immune cells effectively initiate and steer the fracture healing process, their interplay with bone-forming cells is essential for the ultimate outcome of healing. Mesenchymal precursor cells or MSCs are essential for cellular differentiation and tissue restoration since they are the foundation for the formation of a replacement collagen network^59,60^. Murine MSCs can be defined by Sca-1 marker expression on lineage-negative cells (CD45-CD11b-CD34-CD31-Ter119-), and several subtypes have been proposed, with distinct priming processes, functionality, and commitment behavior^61–64^. The immunological experience resulted in alterations in this crosstalk, as the distribution of MSC subtypes differed between the less-experienced (aged) and experienced (immunoaged) groups. The less-experienced group showed a larger increase in the amount of MSCs locally at the fracture site during the initial inflammatory phase, three days post-surgery. The immunoaged group showed, however, higher numbers of skeletal stem cells (mSSC, CD51 expression on the cell surface) and decreased numbers of PDGFRα-expressing MSCs (PαS cells, CD140a expression on the cell surface) among the MSCs at the fracture site. Both populations perform different functions, as mSSCs produce higher levels of collagen type 2 alpha 1 (Col2a1) but also produce higher levels of matrix metalloproteinase 13 (MMP13) than PαS cells. PαS cells display a potent proliferative and self-renewal capacity and robust differentiation potential for all three mesenchymal lineages^65–67^. MSCs change their phenotype and behavior under the influence of an inflammatory environment, and tight regulation of inflammatory cytokines is indispensable for successful healing after injury^68^. This study showed that MSCs are clearly dependent on immune composition and the immune response/signaling. As a consequence of the differing inflammatory signaling patterns, mesenchymal stromal cells were substantially impacted by increased adaptive immune experience in the aged mice, changing the behavior pattern of those cells that are considered to be essential for bone healing and thus impacting healing success.

All cellular changes in phenotype and function alter the local inflammatory milieu and the systemic levels of cytokines. Secreted cytokines are difficult to measure locally; therefore, most studies measure gene expression levels locally, and here, we wanted to analyze the systemically secreted levels of cytokines and establish an intraindividual link between cytokine secretion and immune cell populations, which would not be possible if the fracture tissue had to be used for gene expression analysis. Analysis of systemic cytokine levels at three different timepoints allowed us to study the temporal resolution of inflammatory events during bone regeneration. Increased systemic levels of TNFα and IL-6 in the presurgery state of immunoaged mice indicated a low-grade inflammatory state, which is commonly referred to as inflammaging^24^. IL-6 is widely acknowledged to play a major role during fracture healing, and they have distinct effects on osteoblastic as well as osteoclastic activity by exerting both pro-inflammatory and anti-inflammatory effects^69^. IL-6 neutralization during the initial inflammatory phase or under knockout conditions reduced systemic inflammation and the recruitment of immune cells to the fracture site but significantly delayed the regenerative cascade thereafter^70–72^. IL-6, on the other hand, is responsible for the rapid induction of effector cytokine expression in CD8+ T cells, with known negative effects on fracture healing outcomes^15,73^. IL-6 was found to be upregulated in both groups in this study post-surgery.

Because we found differences in the expression of GM-CSF and IL-22 during bone healing that were dependent on immune experience, these cytokines were tested intensively in vitro in regeneration-related assays in this study. GM-CSF application in vivo rapidly induces the proliferation of mononuclear cells in the bone marrow, and GM-CSF is a key factor in macrophage and dendritic cell maturation, especially for macrophage polarization toward M1^74,75^. Interestingly, T cells produce GM-CSF after activation, recently depicted as a RORγt-dependent activation pathway^76^, and increased GM-CSF levels might prolong the inflammatory state during fracture healing by increasing the maturation and proliferation of dendritic cells and macrophage M1 polarization. In this study, GM-CSF further showed direct interference with stromal and endothelial progenitor cells and negatively affected their regenerative capacity.

This study unravels the role of IL-22 signaling as a core mechanism differentiating healing success in aged and inflammaged mice. IL-22 is produced under inflammatory conditions by activated T cells, mainly by T helper 22 (T_H_22), T_H_17, and T_H_1 cells. The targets of IL-22 are predominantly cells of the nonhematopoietic lineage. IL-22 induces inflammatory mediators in target cells or potentiates the influence of TNFα and/or IL-17-induced pro-inflammatory cytokine expression. In bone regeneration, the role of IL-22 is controversial. Monasterio et al. and Díaz-Zúniga et al. showed a correlation between IL-22 levels and alveolar bone resorption and osteoclast resorptive activity during periodontitis. El-Zayadi et al. showed the contribution of IL-22 to new bone formation in spondyloarthropathies^77–79^. In the present study, IL-22 was proved to be involved in the regenerative process, as shown by in vitro assays, with reduced migratory or proliferative behavior of MSCs as well as reduced tube numbers and branching of de novo formed blood vessels of endothelial cells. However, although IL-22 induced mineralization of the extracellular matrix in vitro, the osteogenic differentiation stimulation cocktail included dexamethasone, which may significantly interfere with the inflammatory response of MSCs. The findings of the present study are in accordance with those of El-Zayadi et al., who showed the effect of pro-inflammatory stimulation of MSCs and the subsequent increase in IL-22 levels. In the present study, we did not pretreat the MSCs with pro-inflammatory stimuli. However, progenitor cells are exposed in vivo to an inflammatory milieu rather than a single cytokine, and the potentiating or inhibitory effects of the cytokines cannot be mirrored directly in vitro.

Finally, to verify the central role of IL-22 as a discriminator of successful and delayed healing in aged patients, we performed a proof-of-concept study by neutralizing or supplementing IL-22 in early fracture healing. Our findings illustrate that IL-22 is a core cytokine differentiating aged and immunoaged healing. Neutralization of IL-22 in immunoaged mice-which showed increased levels of IL-22 post-surgery-induced a healing process comparable to that in less-experienced aged mice (rescue/rejuvenation). Overexpression of IL-22, however, almost completely abolished the regenerative process in more naïve aged mice. Even though some bone formation could be observed directly at the fracture ends, no callus formation in the fracture gap was found. Considering our in vitro results, we deduced that overexpressing IL-22 inhibited de novo vessel formation and hindered progenitor cell migration.

In conclusion, we have identified distinct and specific alterations in the composition and activity of the experienced immune system during the course of regeneration. We showed specific immune cell phenotype changes and subsequently altered cytokine patterns in an advanced animal model that can mimic healing in immunoaged/inflammaged patients. Adaptive immune experience altered the cellular composition locally at the fracture site, as well as the phenotypes and activation states of immune cells and progenitor cells. Reduced healing capacity is linked to the age-associated challenges of fracture patient treatment. Novel factors were identified that may be used for future therapeutic strategies to treat immunologically challenged fracture patients. IL-22 levels were shown to be significantly increased post-surgery under immunologically experienced conditions, and the neutralization of IL-22 reversed the diminished healing capability. Future studies are needed to reveal the potential of immunomodulatory agents to revert and rejuvenate the regenerative process in aged mice, and this study proposes a candidate target.

## Supplementary information

Supplementary Material
