# Supplementary material for: Local immune cell contributions to fracture healing in aged individuals – A novel role for interleukin 22
Source: Exp Mol Med. 2022 Aug 26;54(8):1262–76. doi: 10.1038/s12276-022-00834-9 (PMC9440089; doi:10.1038/s12276-022-00834-9)
Supplement: Supplementary file 1 — Supplementary Material [file 12276_2022_834_MOESM1_ESM.pdf]

Supplementary Material

1 Flow cytometric panels

Supplementary Table 1. Flow cytometric panels 1-4 with the respective markers and clones. All panels included a Live/Dead Fixable Blue stain for ultraviolet excitation (ThermoFisher, Waltham, MA, USA).

Panel 1

marker	*clone*	*supplier*
CD45	30-F11	BD Biosciences, San Jose, CA, USA
CD3e	145-2C11	BD Biosciences, San Jose, CA, USA
CD4	GK1.5	BioLegend, San Diego, CA, USA
CD8a	53-6.7	BioLegend, San Diego, CA, USA
CD335	29A1.4	BioLegend, San Diego, CA, USA
CD11c	N418	BioLegend, San Diego, CA, USA
CD45R/B220	RA3-6B2	ThermoFisher, Waltham, MA, USA
CD62L	MEL-14	BD Biosciences, San Jose, CA, USA
CD44	IM7	BioLegend, San Diego, CA, USA
CD127	SB/199	BioLegend, San Diego, CA, USA
KLRG1	2F1/KLRG1	BioLegend, San Diego, CA, USA
CD205	DEC-205	BD Biosciences, San Jose, CA, USA

Panel 2

marker	*clone*	*supplier*
CD45	30-F11	BD Biosciences, San Jose, CA, USA
CD3e	145-2C11	BD Biosciences, San Jose, CA, USA
CD4	GK1.5	BioLegend, San Diego, CA, USA
CD8a	53-6.7	BioLegend, San Diego, CA, USA
CD44	IM7	BioLegend, San Diego, CA, USA
CD62L	MEL-14	BD Biosciences, San Jose, CA, USA
CD27	LG.3A10	BioLegend, San Diego, CA, USA
CXCR3 (CD183)	CXCR3-173	BioLegend, San Diego, CA, USA
CD43	1B11	BioLegend, San Diego, CA, USA
CD137	17B5	BioLegend, San Diego, CA, USA
PD-1 (CD279)	29F.1A12	BioLegend, San Diego, CA, USA
CD107a	1D4B	BioLegend, San Diego, CA, USA

Panel 3

marker	*clone*	*supplier*
CD45	30-F11	BioLegend, San Diego, CA, USA
CD34	RAM34	BD Biosciences, San Jose, CA, USA
CD11b	M1/70	ThermoFisher, Waltham, MA, USA
CD31	390	BioLegend, San Diego, CA, USA
CD29	HMB1-1	BioLegend, San Diego, CA, USA
CD44	IM7	BD Biosciences, San Jose, CA, USA
Sca-1	D7	BioLegend, San Diego, CA, USA
CD51 (αV Integrin)	RMV-7	BD Biosciences, San Jose, CA, USA
CD140a (PDGFRα)	APA5	BioLegend, San Diego, CA, USA
CD24	M1/69	BioLegend, San Diego, CA, USA

Panel 4

marker	*clone*	*supplier*
CD45	30-F11	BioLegend, San Diego, CA, USA
CD11c	N418	BioLegend, San Diego, CA, USA
CD11b/Mac-1	M1/70	BioLegend, San Diego, CA, USA
CD68	FA-11	BioLegend, San Diego, CA, USA
MHC class II	M5/114.15.2	BioLegend, San Diego, CA, USA
CD80	16-10A1	BioLegend, San Diego, CA, USA
CD86	GL-1	BioLegend, San Diego, CA, USA
CD163	TNKUPJ	ThermoFisher, Waltham, MA, USA
VEGF	VG1	Novus Biologicals, Littleton CO, USA
CD206	C068C2	BioLegend, San Diego, CA, USA

2 Reactive oxygen species (ROS)

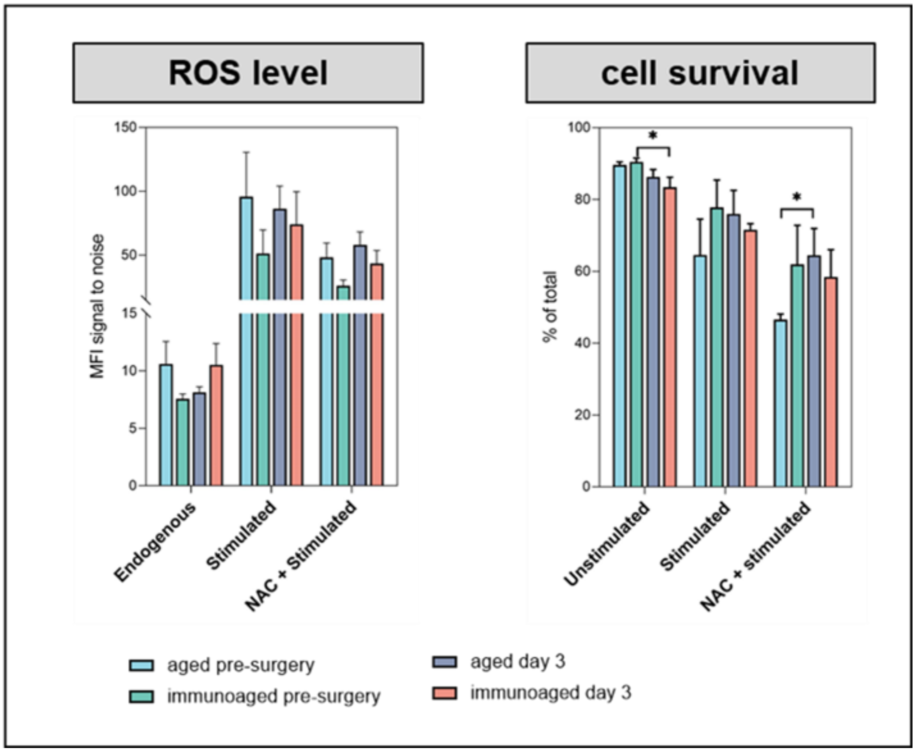

Supplementary Fig. 1. Intracellular reactive oxygen species (ROS) staining in bone marrow and hematoma cells. Hematoma and adjacent bone marrow were isolated and processed with the CellROX Kit (ThermoFisher Waltham, MA, USA) to assess the intracellular levels of ROS. At the pre-surgery level, the aged but immunologically more naïve group showed slight elevated levels of ROS, but in the hematoma phase, three days post-surgery, the aged and immunologically experienced group showed slightly increased levels of ROS. ROS was further induced with tertbutyl hydroperoxide (TBHP) (=stimulated) and inhibited with N-Acetylcysteine (NAC) in combination with TBHP (=NAC + stimulated). No statistically significant differences could be observed between the groups. Cell survival was assessed with Sytox staining (ThermoFisher, Waltham, MA, USA) and showed decreased cell survival when stimulated with TBHP and the combination with NAC.

3 Cytokines and Chemokines

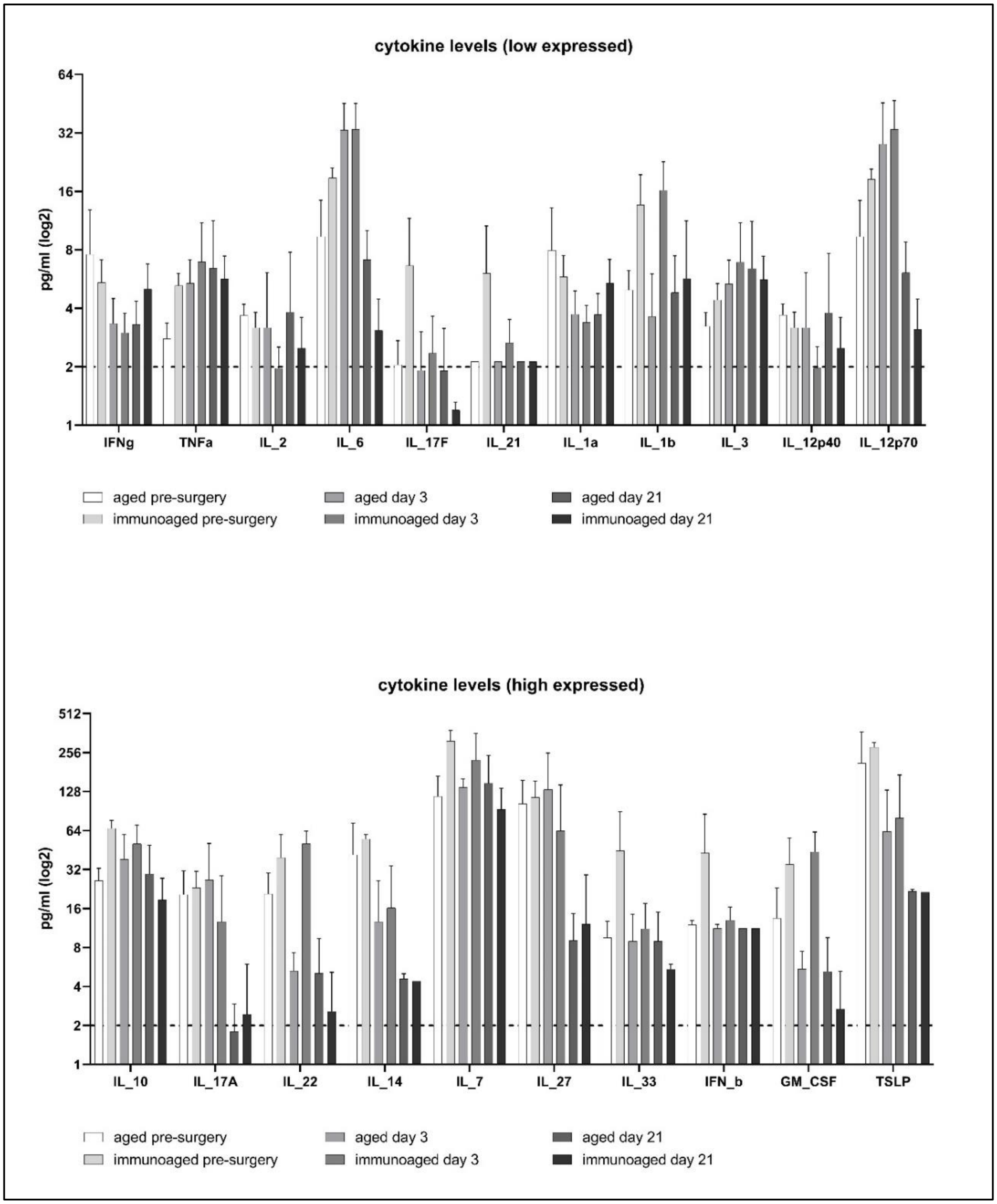

Supplementary Fig. 2. Cytokine levels measured at three different timepoints, pre-surgery, three days post-surgery and 21 days post-surgery. The Cytokine levels were measured via a multiplexed immunosorbent assay (LegendPlex, BioLegend, San Diego, CA, USA). The Cytokines were grouped according their secretion levels into low expression and high expression levels. Dotted line represents the detection limit.

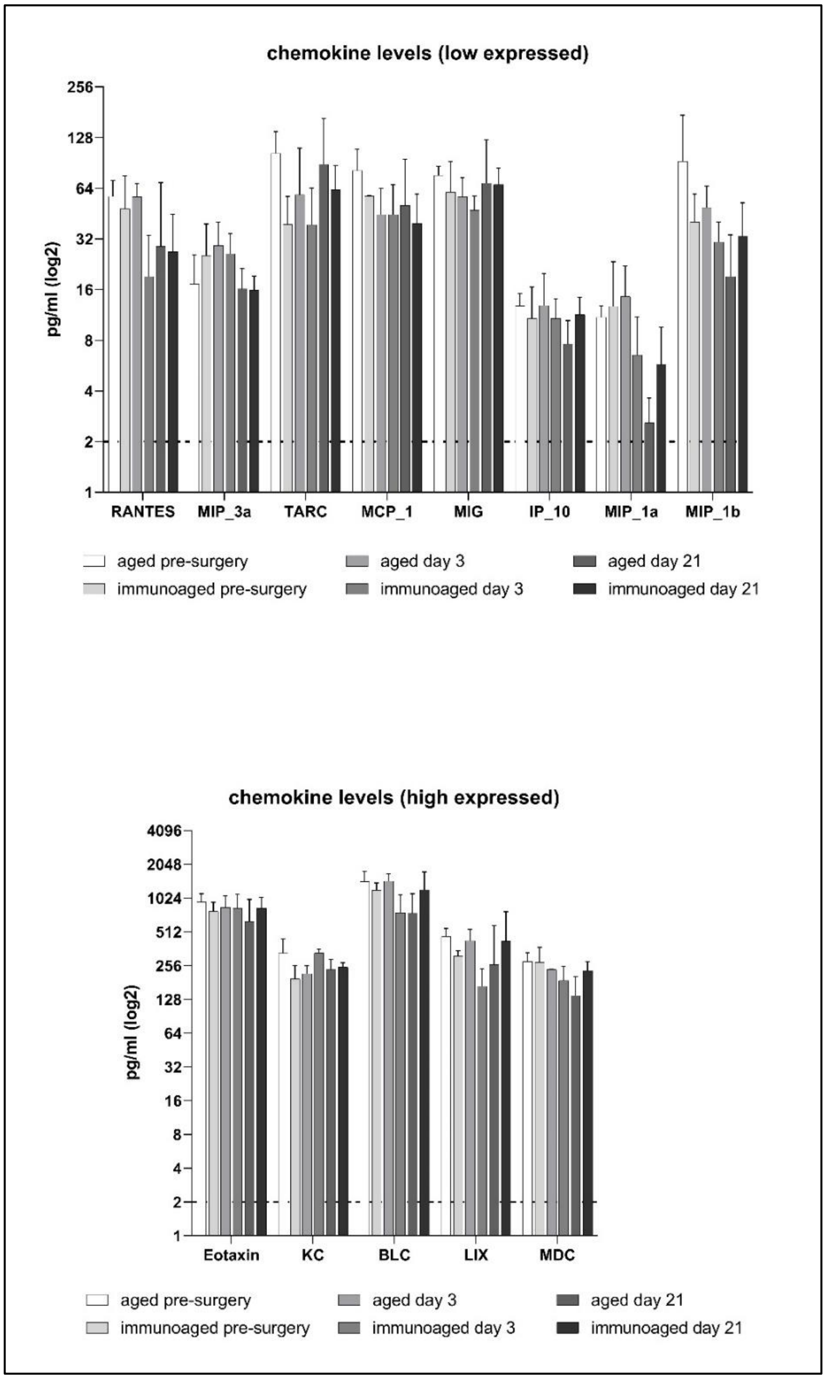

Supplementary Fig. 3. Chemokine levels measured at three different timepoints, pre-surgery, three days post-surgery and 21 days post-surgery. The Cytokine levels were measured via a multiplexed immunosorbent assay (LegendPlex, BioLegend, San Diego, CA, USA). The Chemokines were grouped according their secretion levels into low expression and high expression levels. Dotted line represents the detection limit.

4 Tube formation assay

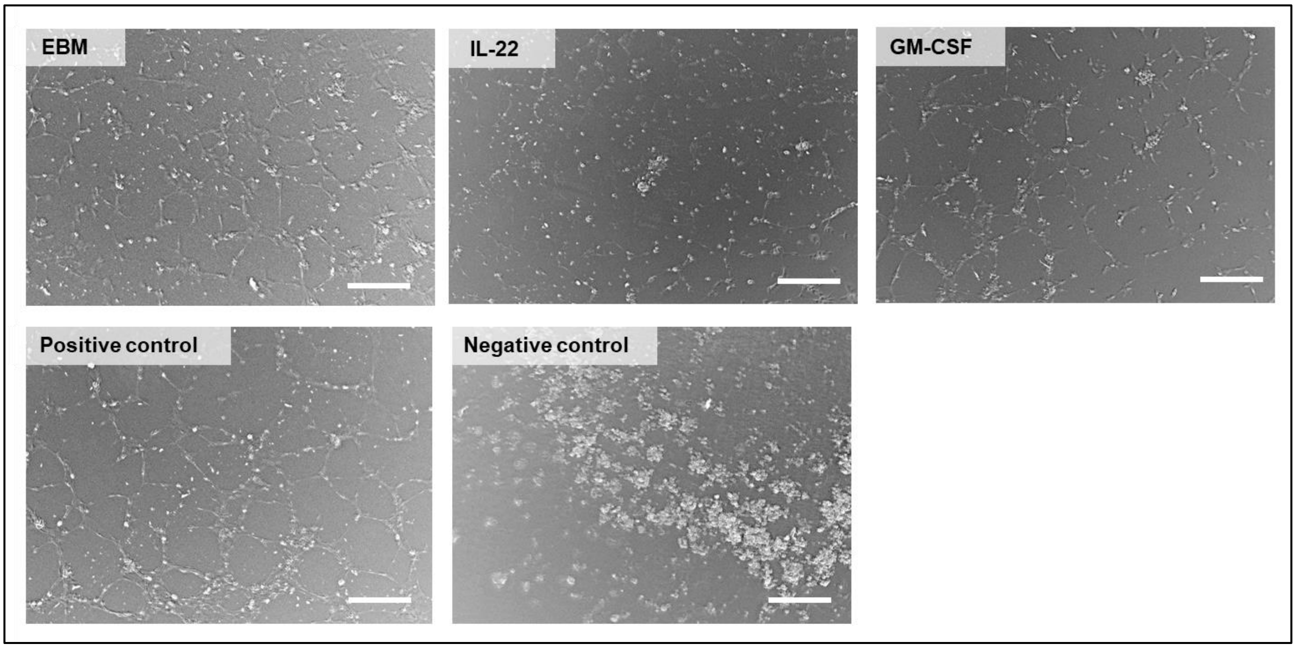

Supplementary Fig. 4. Representative images for the tube formation assay. Human umbilical vein endothelial cells were stimulated with either IL-22 or GM-CSF and compared to the Basal Medium (EBM) and a positive as well as a negative control to assess the degree of tube formation in vitro. IL-22 showed decreased branching points and a decreased number of newly formed tubes. GM-CSF showed only a slight decrease in the tube forming capacity of HUVECS.

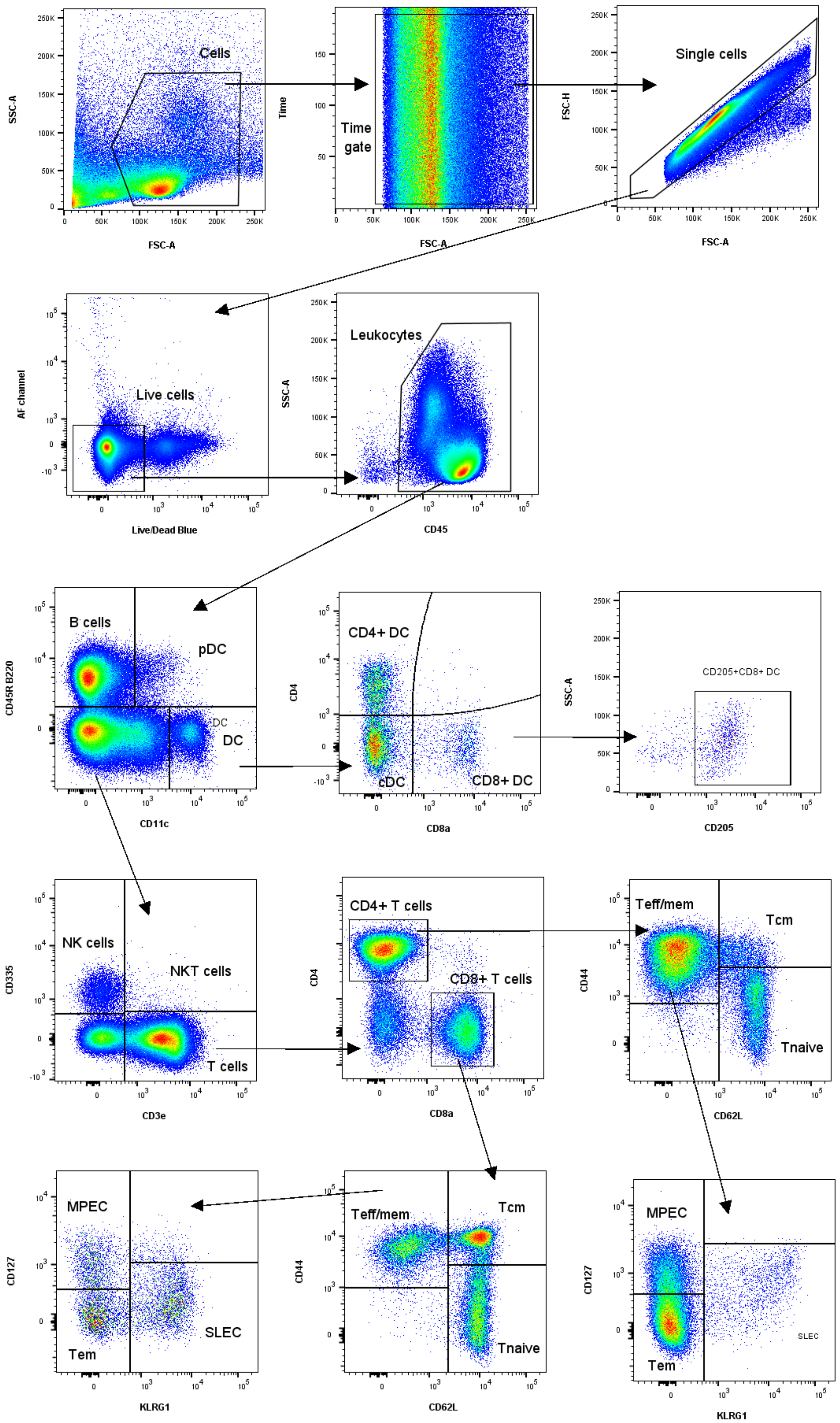

Supplementary Fig. 5 Gating strategy for flow cytometry panel 1. Source material: splenocytes

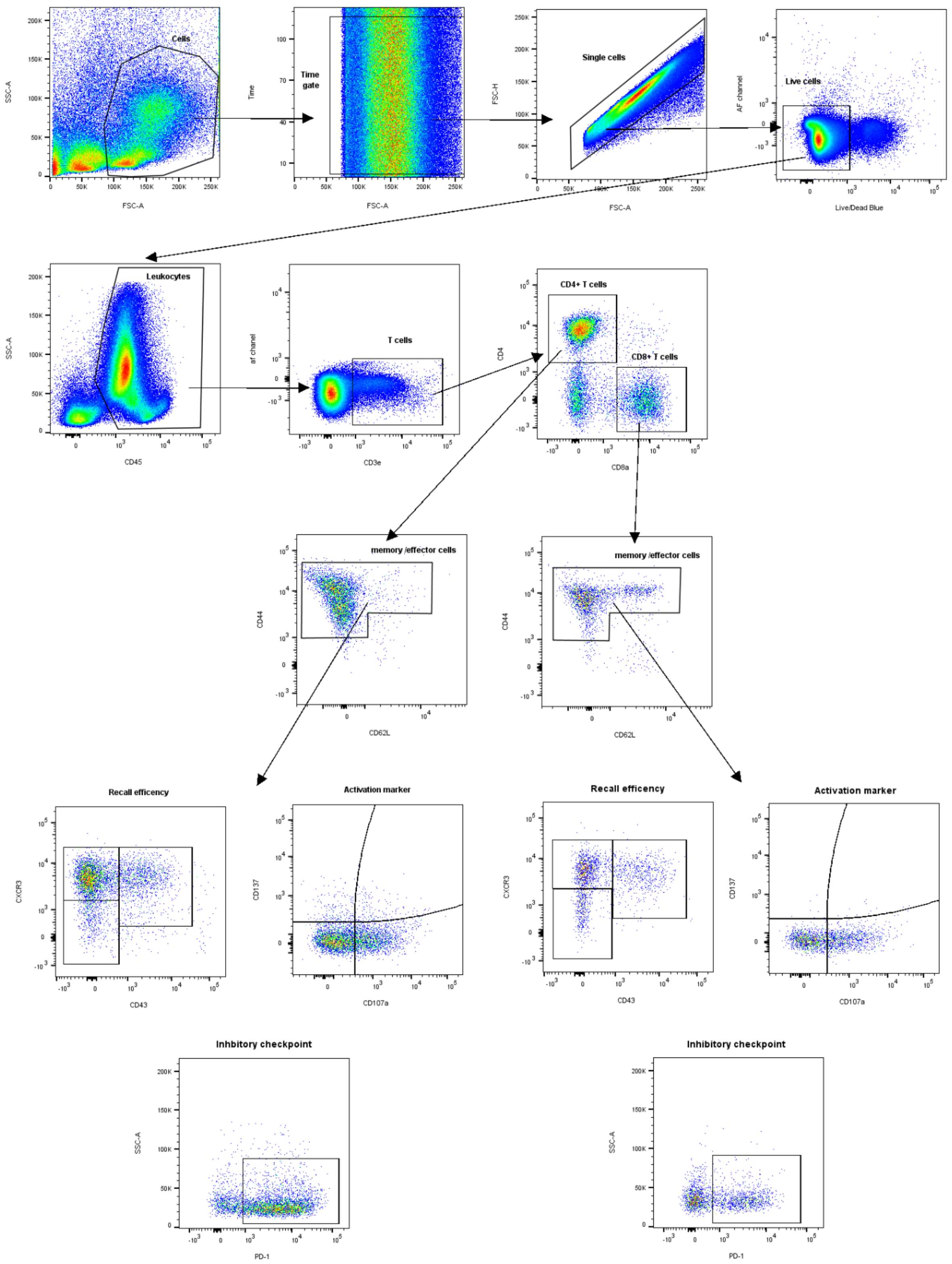

Supplementary Fig. 6 Gating strategy for flow cytometry panel 2. Source material: hematoma

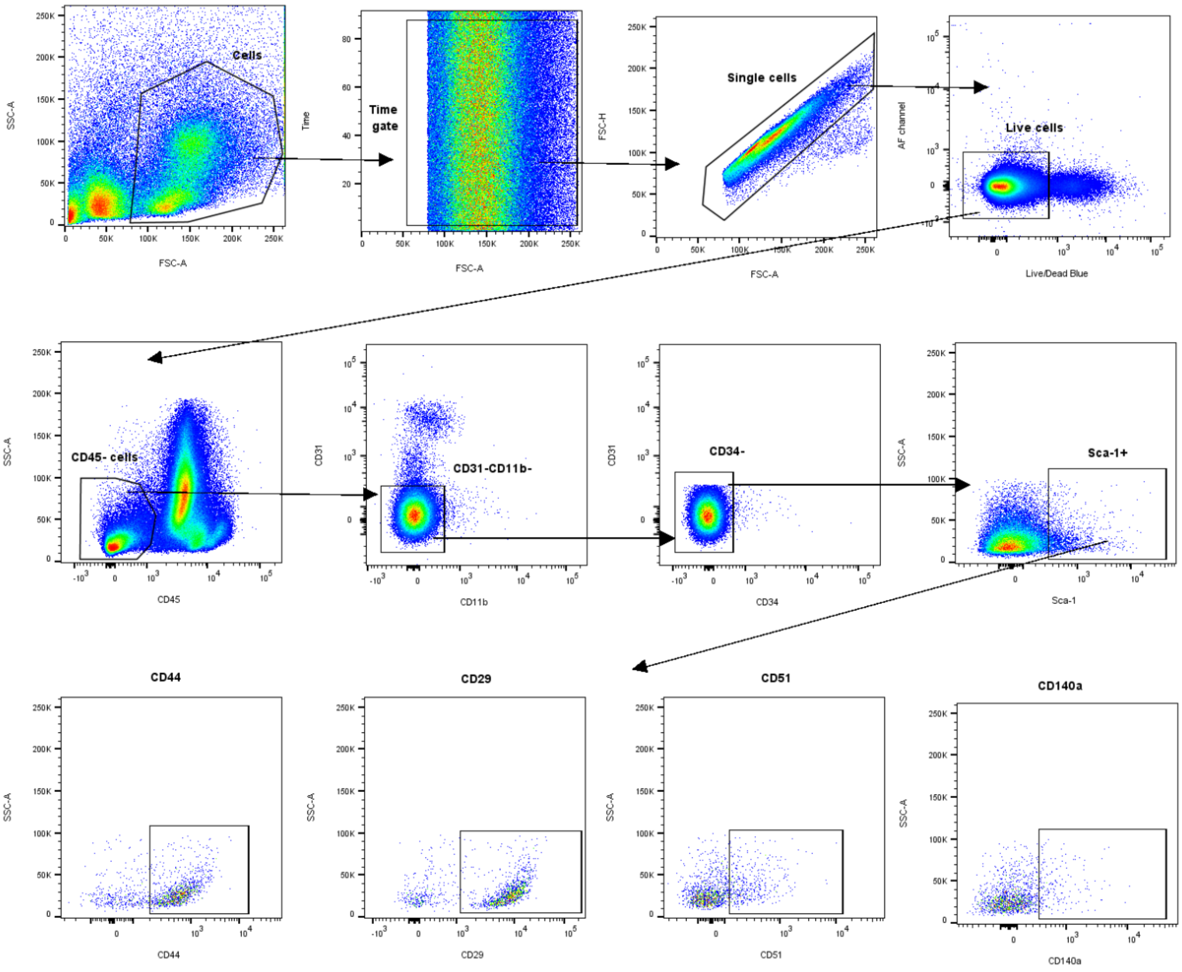

Supplementary Fig. 7 Gating strategy for flow cytometry panel 3. Source material: hematoma

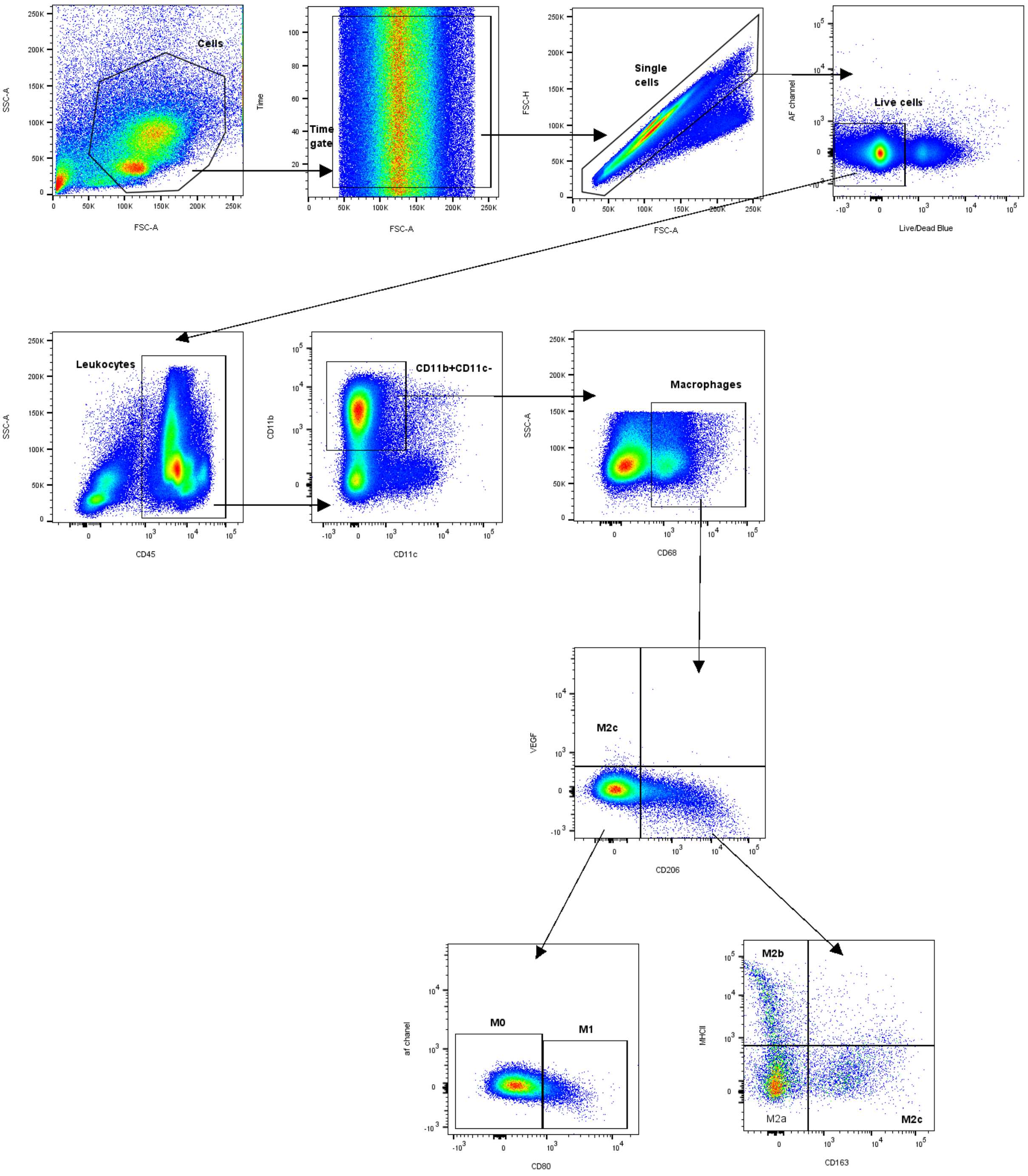

Supplementary Fig. 8 Gating strategy for flow cytometry panel 4. Source material: hematoma
